# Luminescence Fingerprint of Intracellular NIR-II Gold Nanocluster Transformation: Implications for Sensing and Imaging

**DOI:** 10.1021/acsnano.4c13955

**Published:** 2025-02-24

**Authors:** Marina París Ogáyar, Zeineb Ayed, Veronique Josserand, Maxime Henry, Álvaro Artiga, Livia Didonè, Miriam Granado, Aida Serrano, Ana Espinosa, Xavier Le Guével, Daniel Jaque

**Affiliations:** † Nanomaterials for BioImaging Group (nanoBIG), Facultad de Ciencias, Departamento de Física de Materiales, 16722 Universidad Autónoma de Madrid, 28049 Madrid, Spain; ‡ INSERM U1209, CNRS UMR5309, Institute for Advanced Biosciences, 27015 University Grenoble Alpes, F-38000 Grenoble, France; § Nanomaterials for BioImaging Group (nanoBIG), Facultad de Medicina, Departamento de Fisiología, 16722 Universidad Autónoma de Madrid, 28029 Madrid, Spain; ∥ 119906 Instituto de Cerámica y Vidrio | CSIC, Campus de Cantoblanco, 28049 Madrid, Spain; ⊥ Instituto de Ciencia de Materiales de Madrid | CSIC, Campus de Cantoblanco, 28049 Madrid, Spain; # Institute for Advanced Research in Chemical Sciences (IAdChem), Universidad Autónoma de Madrid, 28034 Madrid, Spain

**Keywords:** gold nanoclusters, luminescence, second biological window, intracellular spectroscopy, infrared in vivo imaging, sensing

## Abstract

Gold nanoclusters emitting in the second biological window (NIR-II-AuNCs) have gained significant interest for their potential in deep-tissue bioimaging and biosensing applications due to the partial transparency and reduced autofluorescence of tissues in this spectral range. However, the limited understanding of how the biological environment affects their luminescent properties might hinder their use in bioimaging and biosensing. In this study, we investigated the emission properties of NIR-II-AuNCs when interacting and internalizing into live cells including macrophages, fibroblasts, and cancer cell lines, revealing substantial alterations in their luminescence. A systematic comparison between control and in vitro experiments concluded that the disruption of surface ligands is the main factor responsible for these alterations. NIR-II-AuNCs within cellular environments may also be influenced by other interactions, including aggregation or complexation with proteins. Furthermore, we also corroborated these spectroscopic modifications at the in vivo level, providing additional evidence of the environmental sensitivity of NIR-II-AuNCs. The results obtained in this study contribute to a deeper understanding of the luminescence mechanisms of NIR-II-AuNCs in biological environments in cells and in living tissues and are crucial for their optimization as reliable tools in biological environment for in vitro and in vivo imaging and diagnostics.

## Graphical abstract

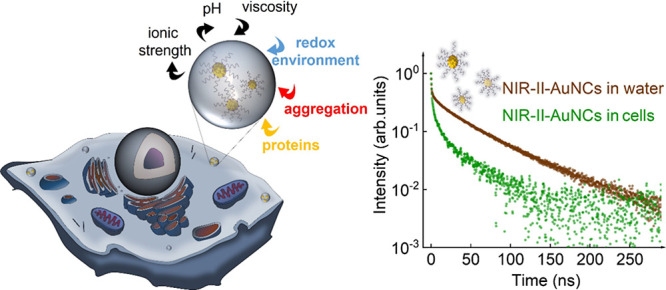

## Introduction

The field of preclinical fluorescence imaging has undergone a significant transformation over the past decade, largely due to the emergence of fluorescent probes operating in the second biological window (NIR-II).^1–3^ The spectral range (1000–1450 nm) is characterized by a unique optical property, where tissues exhibit partial transparency with reduced autofluorescence, resulting from a simultaneous reduction in both scattering and absorption coefficients. Within the NIR-II range, the effective attenuation coefficient of tissues can be as low as 10 cm^–1^ (whereas it can be one or two orders of magnitude larger in the visible), enabling deeper tissue penetration (1–2 cm) and minimal dispersion of infrared light.^4,5^ These attributes have positioned NIR-II luminescent probes as exceptional tools for acquiring high-resolution, deeply penetrating in vivo images at the preclinical level.^6,7^

Pioneering studies have demonstrated the vast potential of NIR-II probes across various applications, including high-resolution whole-body anatomical imaging, vascular imaging, tumor detection, and image-guided surgery.^8–13^ Moreover, certain NIR-II probes display sensitivity to environmental conditions such as temperature, chemical composition, and pressure, allowing their emission characteristics to be leveraged for minimally invasive diagnostics. These probes have been successfully employed in both in vitro and in vivo diagnostics, enabling real-time monitoring of brain activity, early tumor detection, and the diagnosis of inflammation, brain injuries, and liver diseases.^14–23^ The promising capabilities of NIR-II probes have fueled significant research and development efforts aimed at optimizing their physicochemical and luminescent properties, while taking into account the requirements of gram-scale production and safety for possible clinical translation.

Among the various NIR-II probes developed in recent years, gold nanoclusters (NIR-II-AuNCs) have emerged as particularly promising for biosensing and bioimaging applications due to their low cytotoxicity, high photostability, relatively high luminescent quantum yield (QY), high Stokes-shift, and high absorption cross-section to be used as photothermal agents.^24–32^ Additionally, the photoluminescent (PL) emission properties of NIR-II-AuNCs, such as the peak emission wavelength, can be finely tuned by adjusting their composition, core size, and ligand engineering.^20,25,33–35^ For all these reasons, NIR-II-AuNCs have already been employed for high-penetration, high-resolution in vivo imaging in preclinical studies.^36–40^

Despite considerable progress, the mechanisms underlying the NIR-II emission of NIR-II-AuNCs are not yet fully understood.^28,41^ It is documented that their infrared luminescence arises from a complex interplay between the gold core and the surface ligands, making the luminescent properties of NIR-II-AuNCs highly dependent on parameters such as particle size, metal valence state, the nature of surface ligands, and crystallinity.^42,43^ The critical role of surface ligands commanding luminescence means that the properties of NIR-II-AuNCs are also sensitive to environmental conditions, effectively converting them into NIR-II sensing probes.^29,44^ While visible-emitting AuNCs have already been used for intracellular sensing of parameters such as temperature, ions, and reactive species, the potential of NIR-II emitting AuNCs for these applications remains largely unexplored.^45–48^

In this study, we systematically investigated the effect of biological environments on the PL emission properties of NIR-II-AuNCs. Through a comparative analysis of decay curves and PL emission spectra of NIR-II-AuNCs in aqueous solutions and within live cells, we sought to gain a deeper understanding of how biological environments influence their optical behavior. To further elucidate the origins of cell-induced changes in the spectroscopic properties of NIR-II-AuNCs, we conducted control experiments evaluating the effects of various environmental factors, including pH, viscosity, ionic strength, complexation with proteins, aggregation, the disruption of surface ligands, and temperature. Additionally, in vivo hyperspectral imaging experiments were performed to assess the spectral stability of NIR-II-AuNCs following intravenous administration, providing valuable insights into their reliability as optical probes in complex biological settings.

## Results and Discussion

### Spectroscopic Characterization of Colloidal NIR-II-AuNCs in Aqueous Solution

These NIR-II-AuNCs, protected by the two coligands 6-mercaptohexanoic acid (MHA) and hexa­(ethylene glycol) (HDT) molecules, have been described elsewhere^30,35,49^ and exhibit a mean size of 1.5 ± 0.3 nm, measured by transmission electron microscopy (TEM) (Figure S1). Polyacrylamide gel electrophoresis (PAGE) measurements and mass spectrometry characterization confirmed the presence of NIR-emitting atomically precise species with a gold core containing 13–22 atoms (Figures S2 and S3). For the investigation of their fluorescent properties in an aqueous environment, we prepared a solution of NIR-II-AuNCs in distilled water at a concentration of 1 mg/mL. The extinction spectra of NIR-II-AuNCs are constituted by a broadband allowing for optical excitation in the 600–1200 nm range (Figure 1A). Previous studies have associated the NIR absorption of such NCs with energy transitions occurring both within the core and the first ligand shell.^30,35^ The PL emission of NIR-II-AuNCs under 690 nm excitation consists of a broadband emission with a maximum at 1160 nm (Figure 1B). This PL emission results from a combination of quantum confinement effects in the core (d-sp transitions) and surface interactions (ligand-to-metal or ligand-to-metal–metal energy transfer, see Figure 1C).^13,50–54^ Due to the interplay of confinement and interaction effects, the decay curves of NIR-II-AuNCs suspended in water show multiexponential shapes (Figure 1D). Typically, the decay curves of NIR-II-AuNCs are described by a three-exponential function:

$$I(t)=A_{1}\times \exp\left(-\frac{t}{\tau_{1}}\right)+A_{2}\times \exp\left(-\frac{t}{\tau_{2}}\right)+A_{3}\times \exp(-\frac{t}{\tau_{3}})$$ 1

where *I* is the NIR emitted intensity, *t* is the time after pulse excitation, and *A*_*i*_ are the amplitudes associated with each decay time τ_i_. The fast decay component (τ_1_ ≤ 4 ns) is typically associated with electronic relaxations within the gold core, whereas the slow components (τ_2_, τ_3_ > 4 ns) are associated with the radiative decays generated by gold shell states, in which a significant ligand contribution is present. From the values of *A*_*i*_ and τ_i_ provided by the fitting, it is possible to calculate the intensity-weighted average lifetime:

$$\overset{®}{\tau }=\frac{1}{W_{t}}=\sum_{i=1}^{3}A_{i}\times \tau_{i}^{2}/\sum_{i=1}^{3}A_{i}\times \tau_{i}$$ 2

where *W*_t_ is the total decay. When the decay curves obtained at different emission wavelengths are analyzed, we found that the average lifetime decreases for longer wavelengths, mainly caused by the reduction in the slow decay time τ_3_ (see Figure 1E).

**Figure 1:**
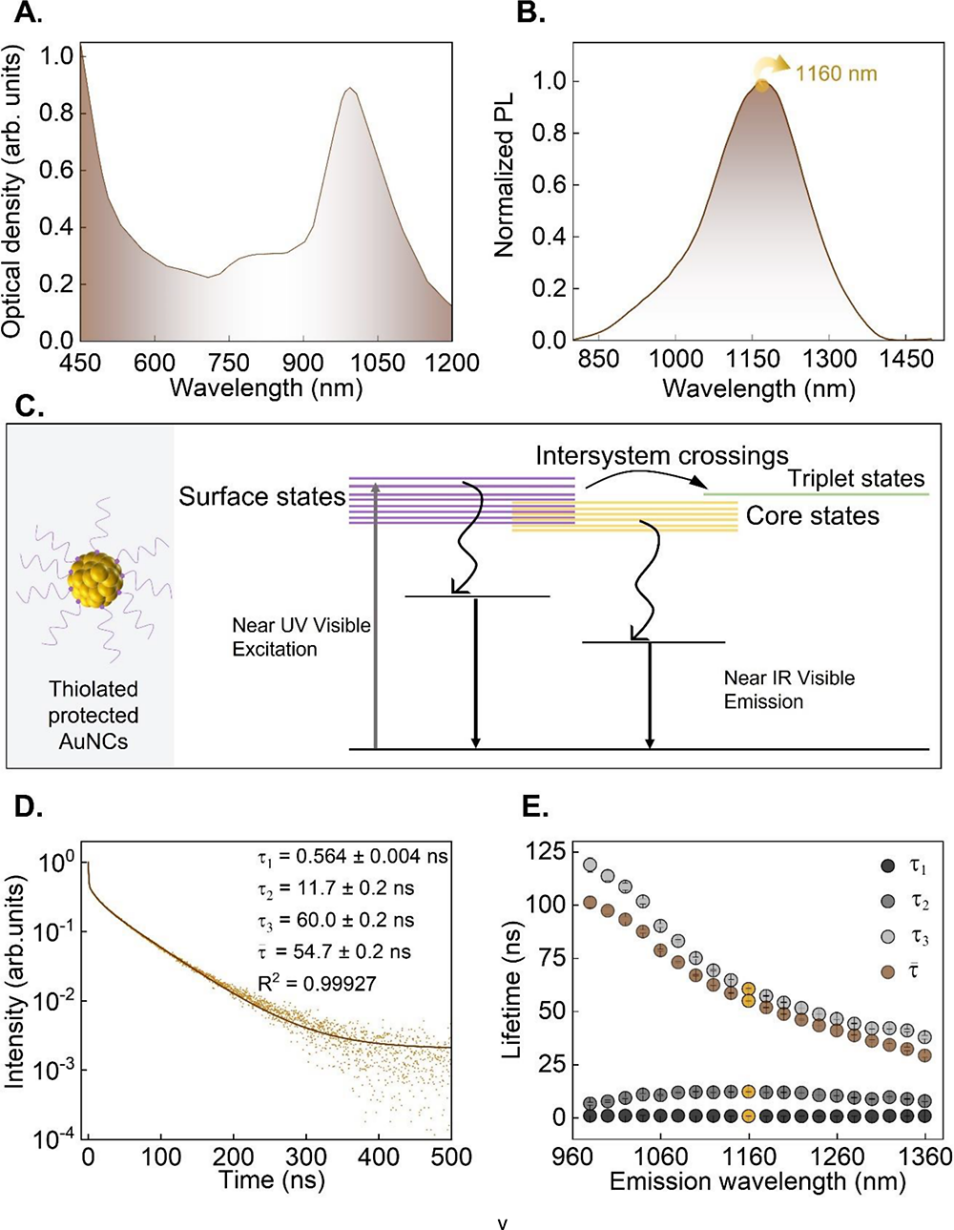
(A) Extinction spectrum of an aqueous solution of NIR-II-AuNCs measured at 25 °C. (B) Emission spectrum generated by NIR-II-AuNCs dispersed in water, as obtained at 25 °C. The excitation wavelength was 690 nm. (C) Schematic representation and energy level diagram of NIR-II-AuNCs. (D) Luminescence decay curve corresponding to NIR-II-AuNCs dispersed in water. Excitation and emission wavelengths were 634.3 and 1160 nm, respectively. Data obtained at 25 °C. Symbols are experimental data, and the solid line is the best fit to a three-exponential decay. (E) Wavelength dependence of the three decay times (τ_1_, τ_2_, and τ_3_) as well as of the average lifetime (τ̅).

### Internalization of NIR-II-AuNCs in Live Cells

To evaluate the luminescence properties of our NIR-II-AuNCs in biological environments, we first evaluated their cytotoxicity and then explored their ability to be internalized by live cancer cells using the Uppsala 87 Malignant Glioma (U87) cell line. Cytotoxicity was evaluated by an MTT assay after 24 h of incubation using NIR-II-AuNC concentrations ranging from 15 to 240 μg/mL. The cytotoxicity assay (Figure S4A) did not reveal significant cellular toxicity in any of the tested concentrations, adhering to the standardized guidelines for the MTT assay in ISO-10993-5, where toxicity is defined as less than 70% viability.

Cellular uptake of the NIR-II-AuNCs for different incubation times can be evidenced by combining NIR-II fluorescence with optical microscopy. Maximum uptake, monitored thorough the signal-to-background ratio of NIR-II cellular images, was observed for an incubation time of 24 h and for a concentration of NIR-II-AuNCs of 100 μg/mL (Figures 2A and S4B). These incubation conditions ensure a high signal-to-noise ratio in both PL emission and lifetime measurements, while ensuring cell viability above 90%. The NIR-II fluorescence images reveal a negligible presence of NIR-II-AuNCs outside the live cells (Figures 2A and S4B). The absence of extracellular NIR-II-AuNCs is essential to perform intracellular spectroscopic characterization as it ensures that all the luminescence signal analyzed is generated by NIR-II-AuNCs within a cellular environment. Endosomal internalization of NIR-II-AuNCs in U87 cells after 24 h of incubation was verified by TEM, with the detection of discrete AuNCs in these amorphous vesicles (Figure 2B). The endocytosis uptake was validated by energy-dispersive X-ray spectroscopy, which confirmed the gold composition of the particles localized in the lysosomes within the cells (Figure S5A). Furthermore, cellular experiments using LysoTracker demonstrated strong colocalization with a Pearson coefficient of *r* = 0.67, providing robust evidence of an endocytic uptake mechanism for these particles in U87 cells (Figure S5B).

**Figure 2:**
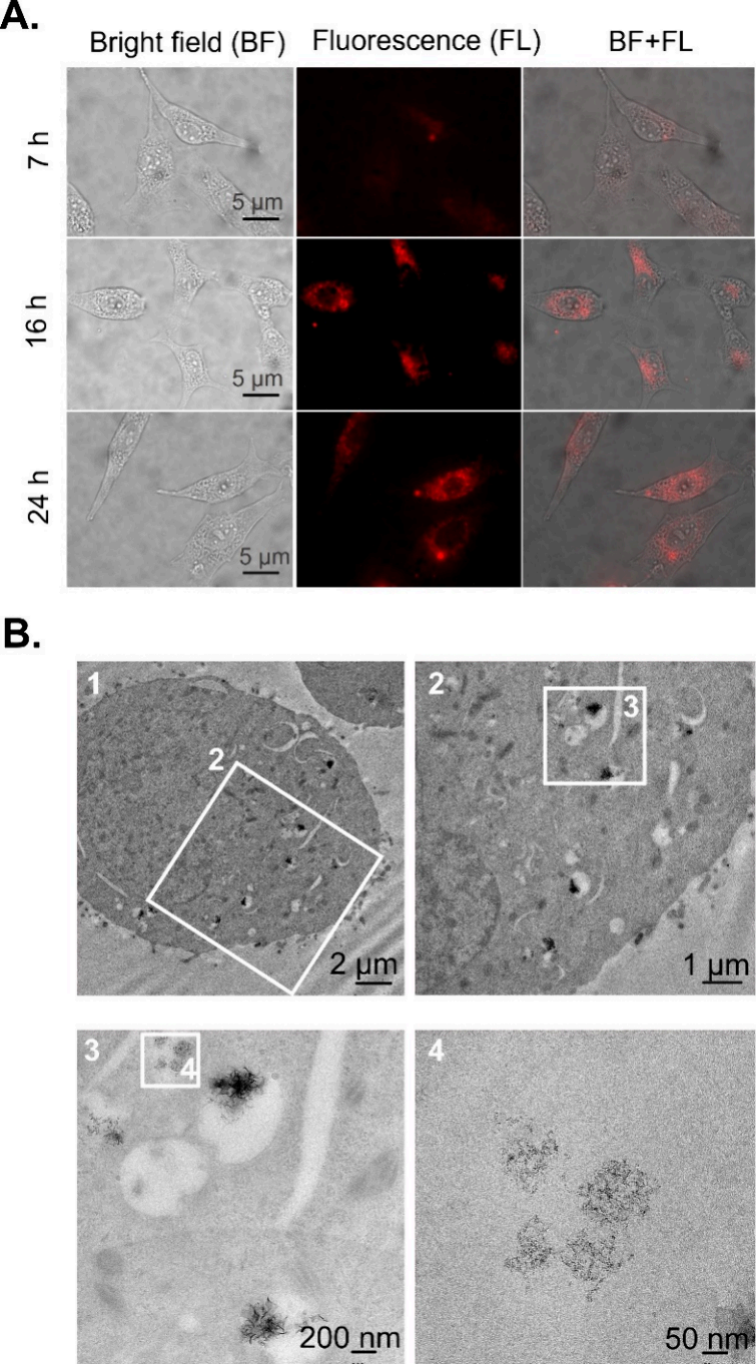
(A) Representative bright field, NIR-II fluorescence, and merged images of U87 cells after incubation with NIR-II-AuNCs (100 μg/mL) during 7, 16, and 24 h. (B) TEM images of a U87 cell at different magnifications after 24 h of incubation with AuNCs at a concentration of 100 μg/mL, revealing their endosomal internalization.

### Intracellular Spectroscopy of NIR-II-AuNCs

For intracellular spectroscopic studies, pellets of U87 cells were collected after 24 h of incubation in a culture medium containing NIR-II-AuNCs at a concentration of 100 μg/mL. The cell pellets were placed inside a transparent cuvette and optically excited with a continuous wave (690 nm laser) for the acquisition of NIR-II emission spectra or with a picosecond pulsed laser at 634.3 nm for the acquisition of decay curves. Under 690 nm excitation, the NIR-II emission from the cell pellet is characterized by a broadband centered at around 1080 nm (Figure 3A). When compared with results obtained in aqueous solutions, the NIR-II emission spectrum of NIR-II-AuNCs has shifted ∼80 nm toward shorter wavelengths. In addition, the presence of NIR-II-AuNCs within cells also caused an increment in the full width at half-maximum (fwhm) of the emission band from 215 up to 320 nm. The spectral distortions evidenced in Figure 3A could be attributed to two phenomena:

i) to the wavelength-dependent extinction of NIR-II emission when crossing the cell pellet.
ii) to the modification in the physical–chemical properties of NIR-II-AuNCs induced during their incorporation into cells.

**Figure 3:**
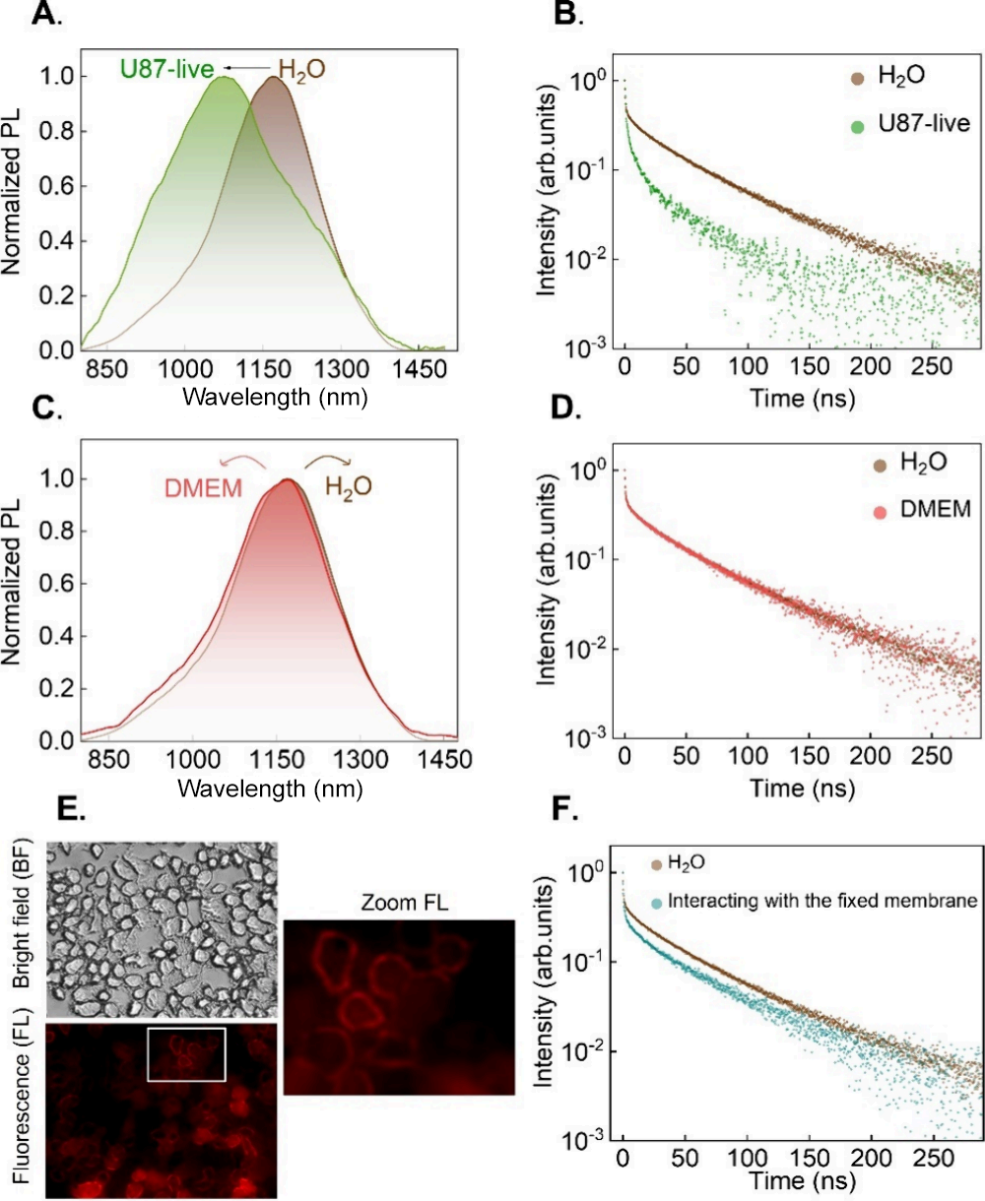
Comparison of the emission spectra (A) and decay curves (B) corresponding to NIR-II-AuNCs dispersed in water and within U87 living cells. Comparison of the emission spectra (C) and decay curves (D) corresponding to NIR-II-AuNCs dispersed in water and in a cell culture medium (DMEM). (E) Optical and NIR-II fluorescence images of U87-fixed cells revealing the presence of NIR-II-AuNCs attached to the cell membrane. (F) Decay curve obtained for NIR-II-AuNCs attached to the cell membrane of U87-fixed cells. All of the data were obtained at 25 °C. The excitation and emission wavelengths for the acquisition of decay curves were 634.3 and 1160 nm in all the cases, respectively.

The cell pellet is a highly scattering medium with an extinction coefficient increasing for short wavelengths (Figure S6). This would lead to a larger attenuation of high-energy (short-wavelength) photons within the emission band of NIR-II-AuNCs. This, in turn, would lead to a redshift of the emission. This is contrary to what was experimentally observed, suggesting that the differences evidenced in Figure 3A are not caused by light extinction within the pellet but, instead, by an actual modification of the spectroscopic properties of NIR-II-AuNCs. The change of the inherent spectroscopic properties of NIR-II-AuNCs when incorporated into cells is further supported by the luminescence decay curves. The luminescence decay curve (λ_exc_ = 634.3 nm, λ_em_ = 1160 nm) recorded for NIR-II-AuNCs within live cells reveals a faster de-excitation of excited electrons with respect to NIR-II-AuNCs in water (see Figure 3B). As it occurs in water, the decay curve of our NIR-II-AuNCs does not follow a single exponential decay and can be fitted to a three-exponential decay (Figure S7). When NIR-II-AuNCs are accumulated within live cells, the average lifetime at 1160 nm of NIR-II-AuNCs decreases from 54.7 ± 0.2 ns in solution down to 32 ± 2 ns within cells. The simultaneous reduction in the relative contribution of slow components and the average lifetime revealed that, during the intracellular incorporation processes, the structure of NIR-II-AuNCs has been affected, leading to the appearance of new nonradiative pathways as well as to a reduction in the relative contribution of de-excitations associated with the ligands.

### Key Intracellular Factors Influencing the Spectroscopic Response of NIR-II-AuNCs

Once the NIR-II-AuNCs are dispersed in the cell culture medium, they start to interact and accumulate on the cell membrane. Next, they are uptaken into cells via cellular mechanisms such as endocytosis.^55^ The uptake mechanism is highly dependent on the size and surface properties of AuNCs.^45,56–60^

Decay curves and PL emission spectra corresponding to NIR-II-AuNCs dispersed in the cell culture medium (Dulbecco’s modified Eagle’s medium, DMEM) show no significant differences to those obtained from NIR-II-AuNCs dispersed in aqueous solution (Figure 3C,D). Thus, we discard the possibility that the electronic modification of NIR-II-AuNCs is being produced because of their interaction with components from the cell culture medium such as proteins or lipids.

To evaluate the nature of the interaction with the cell membrane, we added the NIR-II-AuNCs to a population of prior fixed cells. As illustrated by the NIR-II fluorescence images, the NIR-II-AuNCs are localized at the cell membrane (Figure 3E). The PL decay curves corresponding to NIR-II-AuNCs attached to the membrane revealed a slight modification compared to NIR-II-AuNCs dispersed in aqueous solution, with a reduction in the relative contribution of the ligand interaction component while maintaining the values of the average decay times (Figure 3F and Table S1). This suggests that the interaction with the cell membrane could not explain the main spectroscopic alterations when NIR-II-AuNCs are internalized (Figure 3A,B).

Once the interaction with the cell culture medium and cell membrane are discarded as the main mechanisms causing the alterations in the spectroscopic properties of NIR-II-AuNCs, we explore the spectroscopic changes due to the interaction of NIR-II-AuNCs with the biological environment of the cytoplasm. Distinct cellular compartments can alter the spectroscopic properties of NIR-II-AuNCs through different mechanisms including variations in pH, viscosity, and ionic strength, the induction of aggregation, complexation with proteins, and/or the breakage of surface ligands (Figure 4A).^40,55,61^

**Figure 4:**
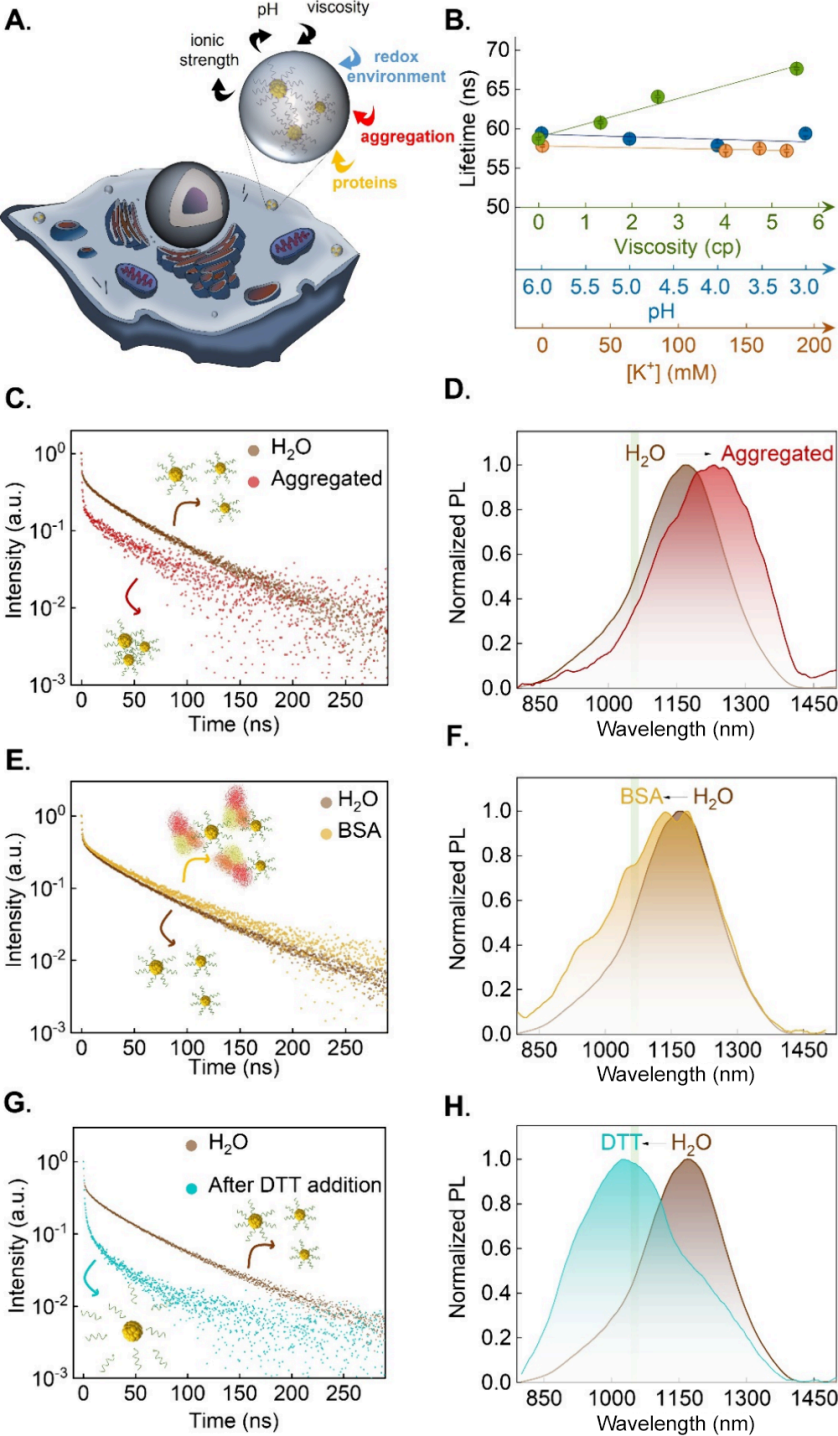
(A) Scheme of a cell including possible parameters that can affect the spectroscopic properties of NIR-II-AuNCs within the cell environment. (B) Average luminescence lifetime of NIR-II-AuNCs as obtained as a function of viscosity (range 0–5.6 cp), pH (range 3.0–6.0), and K^+^ concentration (range 0–180 mM) in the aqueous medium. Data were obtained at 25 °C. Luminescence decay curves (C) and emission spectra (D) were obtained at 25 °C corresponding to NIR-II-AuNCs dispersed in water and after inducing aggregation with polyethylenimine (PEI). Luminescence decay curves (E) and emission spectra (F) obtained at 25 °C correspond to NIR-II-AuNCs dispersed in water after complexation with proteins by the addition of bovine serum albumin (BSA) to the solution. Luminescence decay curves (G) and emission spectra (H) obtained at 25 °C correspond to NIR-II-AuNCs dispersed in water and after the breakage of ligands with dithiothreitol (DTT). The green solid lines in (D,F,H) indicate the maximum emission intensity measured in U87 living cells (1080 nm).

Therefore, we intended to evaluate the spectroscopic properties of NIR-II-AuNC separately for each of these factors:

i) *pH* – Intracellular pH gradients exist across the different subcellular compartments and organelles.^62,63^ While the cytoplasm maintains a neutral pH of approximately 7.2, certain compartments exhibit more acidic pH. Lysosomes, for instance, could show a pH close to 5, while the nucleus and the peroxisomes maintain a slightly alkaline pH (around ≈8). The fluorescence decay curves obtained in solutions with different pH (Figure S8) revealed that the fluorescence decay rate of NIR-II-AuNCs is not significantly affected by the pH of the surrounding medium (range of pH 3–14). Indeed, the average lifetime obtained from the decay curves remained independent of pH (Figures 4B and S9). Thus, we conclude that *pH* is not responsible for the spectroscopic changes revealed by intracellular spectroscopic measurements.
ii) *Viscosity* – The cytoplasm is known to be a medium characterized by highly inhomogeneous viscosity, with average viscosity values well above of water.^64^ To investigate the effect of viscosity, we increased the viscosity in solution by adding different quantities of glycerol. As observed in Figures 4B and S10, increasing viscosity notably prolongs the average fluorescence lifetime of NIR-AuNCs. However, this trend is opposite to our observations in cells (the average lifetime decreases within cells), suggesting that viscosity has probably a minor effect on the spectroscopic properties of NIR-II-AuNCs within cells.
iii) *Ionic strength* – The intracellular ionic strength modulates several cellular functions by promoting the interaction of biomolecules or regulating the enzymatic activity.^65^ Within cells, potassium (K^+^) is the most prevalent cation at a concentration of [K^+^] ≈ 140–150 mM.^66^ Figures 4B and S11A demonstrate that the average fluorescence lifetime of NIR-II-AuNCs remains invariable when evaluating different ionic strengths within the physiological range at different K^+^ concentrations. Additionally, we demonstrate that it remains unchanged upon the addition of primary ion concentrations (Na^+^, K^+^, and Fe^3+^) typically found within cells (Figure S11B).^67–70^

iv) *Aggregation state* – Nanoparticles, particularly when internalized by endosomes, often present rearrangements and aggregation within the cell. Indeed, TEM images depicted in Figure 2B demonstrate that NIR-II-AuNCs tend to accumulate in vesicles within the cytoplasm of U87 cells. To investigate the aggregation behavior and interaction between multiple NIR-II-AuNCs, we employed the cationic electrolyte polyethylenimine hydrochloride (PEI) at a high concentration (60 mM). The decay curves recorded in these conditions reveal that aggregation leads to a significant reduction in the relative contributions of the components related to the ligands (Figures 4C, S12 and Table S1), as it was observed in intracellular located NIR-II-AuNCs (Figure 3B). Furthermore, our results agree with previous works published by L. Haye et al., who demonstrated that encapsulating over 10,000 NIR-II-AuNCs within a 60 nm polymeric structure leads to a reduction in the relative contribution of the slow lifetime component.^34^ Interestingly, in accordance with previous results published by Haye et al., the emission spectrum of aggregated NIR-II-AuNCs revealed a redshift (Figure 4D). This bathochromic shift is contrary to the hypsochromic shift obtained for intracellularly located NIR-II-AuNCs (Figure 3A). Thus, we conclude that intracellular aggregation of NIR-II-AuNCs solely cannot explain the spectroscopic changes induced during intracellular incorporation.
v) *Complexation with biological compounds present in cells* – Proteins play a crucial role in almost every aspect of cellular function. When nanoparticles are internalized by cells, proteins (including albumin, apolipoprotein, or fibrinogen) cover their surface.^71^ To investigate the impact of complexation of different biological compounds or proteins on the spectroscopic properties of our NIR-II-AuNCs, we conducted experiments using bovine serum albumin (BSA) as a model to simulate the complexation of protein around NIR-II-AuNCs within live cells. BSA was added at a high concentration to a solution of NIR-II-AuNCs. While the interaction induces a blueshift in the emission of NIR-II-AuNCs (as it occurs within cells), it also induces an increment in the average lifetime that is contrary to the reduction obtained within live cells (Figure 4E,F and Table S1), as already reported in the literature.^13,72^ This suggests that, as happened with aggregation, complexation with proteins alone cannot explain the spectroscopic properties of intracellularly located NIR-II-AuNCs.
vi) *Cytoplasm-induced breakage of surface ligands* – The cytoplasm of cells is characterized by its reducing nature, which inhibits the formation of disulfide bonds.^73,74^ The redox potential of the cytoplasmatic environment or endolysosomal vesicles can have significant implications for certain structures containing thiol groups, implying ligand exchange or growing bigger gold nanostructures.^75^ Therefore, when NIR-II-AuNCs are accumulated within cells, it is expected that the reducing nature of the cytoplasm could compromise the integrity of surface ligands (MHA and HDT molecules in this work). To check whether the breakage of surface ligands could explain the intracellular behavior of our NIR-II-AuNCs, we designed a control experiment in which we use dithiothreitol (DTT) to induce the breakage of the ligands (breakage of HDT dimer adsorbed on AuNC surface or Au-S bonds, or both). The addition of DTT led to a significant change in the luminescence decay curves consisting in a reduction in the relative contribution of the slow components (Figure 4G). Indeed, we found that the breakage of surface ligands led to similar decay curves to those obtained within living cells (Figure 3B and Table S1). Furthermore, the PL emission spectra obtained after the breakage of surface ligands (Figure 4H) presents a spectral blueshift of 120 nm, which is more significant to that obtained within live cells (80 nm, from Figure 3A).

The experiments described above suggest that pH, viscosity, ion strength, aggregation state, and complexation with proteins are factors that could synergistically modulate the spectroscopic response of NIR-II-AuNCs within cells. Nevertheless, we found that none of them simultaneously account for the blueshift of emission spectra and the reduction in the average lifetime. The behavior observed in cells, in both the emission spectra and decay curves, is better explained by the breakage of the surface ligands, due to the reducing nature of the cytoplasm and lysosomes. Thus, we conclude that the relevant modifications in the spectroscopic parameters of NIR-II-AuNCs when they accumulate within cells are mainly caused by (i) the complexation with biological compounds present in cells, (ii) their aggregation in endosomes, and as the primary reason, (iii) the reducing nature of the cytoplasm, which leads to the breaking of surface ligands.

### Modification of NIR-II-AuNCs in Other Cell Lines

To corroborate whether the observed modifications in the spectroscopic properties of NIR-II-AuNCs are not cell line-specific, we conducted parallel experiments in RAW 264.7 macrophage and 3T3/L1 fibroblast cell lines. Following the same protocol employed for the U87 cell line, NIR-II-AuNCs were incubated for 24 h at a concentration of 100 μg/mL. Fluorescence microscopy confirmed the uptake by both macrophages and fibroblast cells (Figure S13). Emission experiments reveal how in these two cell lines the emission spectra of NIR-II-AuNCs undergo modifications (blueshift) similar to those observed in U87 cells (Figure S14A). However, when the decay curves are analyzed, some differences are noted. A decrease in the averaged lifetime was observed in macrophages, though less pronounced than in U87 cells, whereas NIR-II-AuNCs within the fibroblast retain the decay curve obtained in water (Figure S14B). These results suggest that the modification of the spectroscopic properties of NIR-II-AuNCs due to intracellular accumulation seems to be a universal effect, although the magnitude of the spectral modifications is cell-line-dependent. This, indeed, makes sense as different cell lines may show differences in endocytic pathways, internalization rates, metabolic pathways, and metabolic rates, resulting in different degrees of agglomeration, breakage of surface ligands, and complexation with proteins that may lead to different alterations in the spectroscopic properties of NIR-II-AuNCs.^76–78^

### Implications in Intracellular Sensing with NIR-II-AuNCs

As commented in the Introduction, one of the potential applications of NIR-II-AuNCs is their application as intracellular sensors by using their luminescence for providing information about the intracellular environment. Nevertheless, the multiple dependence of the spectroscopic properties of NIR-II-AuNCs on different parameters (Figure 4) could constitute a limitation for their use due to the appearance of cross-talk: changes in different physiological parameters could have the same signature in the spectroscopic properties of NIR-II-AuNCs. To illustrate this limitation, we, as a case of study, explore the robustness of NIR-II-AuNCs as intracellular thermal sensors, a possibility already demonstrated for visible-emitting AuNCs. The luminescence of NIR-II-AuNCs suspended in water exhibits a temperature-dependent behavior, characterized by a relevant thermal quenching in the physiological temperature range (20–45 °C) that is evidenced by a simultaneous reduction in emitted intensity (Figure 5A,B) and in the average lifetime (Figure 5C,D). The thermal quenching of NIR-II-AuNCs is attributed to a reduction in metal–ligand interactions at high temperatures, which consequently weakens charge transfer processes and introduces novel nonradiative relaxation pathways.^79^ The linear reduction of the average lifetime with temperature (Figure 5D) reveals that NIR-II-AuNCs, when dispersed in water, are capable of remote thermal sensing with a relative sensitivity of *S*_r_(H_2_O, 37 °C) ≅ 1.1% °C^–1^. When incorporated within U87 live cells, the emitted intensity also reduces with temperature, but in comparison to the results obtained in water, in a less pronounced manner (Figure 5A,B). The average lifetime of NIR-II-AuNCs is significantly shorter than in water, and it reduces with temperature, with a thermal sensitivity *S*_r_(U87 live, 37 °C) ≅ 0.23% °C^–1^ that is half the sensitivity obtained for NIR-II-AuNCs in water (Figure S15). The different values of both τ̅ and *S*_r_ obtained in water and within U87 live cells imply that the traditional approach—translating intracellular lifetime values into intracellular temperature by using a calibration (τ̅ vs *T*) curve obtained in an aqueous suspension—is no longer valid. Instead, the use of NIR-II-AuNCs as intracellular thermal reporters would require using a calibration curve obtained in live cells. The use of NIR-II-AuNCs as intracellular thermal reporters becomes even more complicated when their thermal response is also analyzed in fixed cells, i.e., in the absence of cell metabolism. Within fixed cells, the average lifetime of NIR-II-AuNCs lies between the values obtained in live cells and in aqueous suspension (Figure 5D). In the absence of cell metabolism, the average lifetime reduces with temperature with a thermal sensitivity significantly different from those obtained in live cells but closer to the values in water: *S*_r_(U87-fixed, 37 °C) ≅ 1.2% °C^–1^ (Figure S15). The dependence of both τ̅ and *S*_r_ on the presence/absence of metabolic activity discards NIR-II-AuNCs as reliable lifetime-based thermal reporters. We should note that intracellular metabolism has also been identified as a source of bias in other intracellular thermometers including fluorescent proteins or semiconductor quantum dots.^80^ In the particular case of NIR-II-AuNCs, the relevant role of metabolism was expected: cell metabolism induces dynamical changes in the physiological properties of the cytoplasm, and as demonstrated in Figures 3 and 4, this affects the spectroscopic properties of NIR-II-AuNCs (even in the absence of any temperature variation).

**Figure 5:**
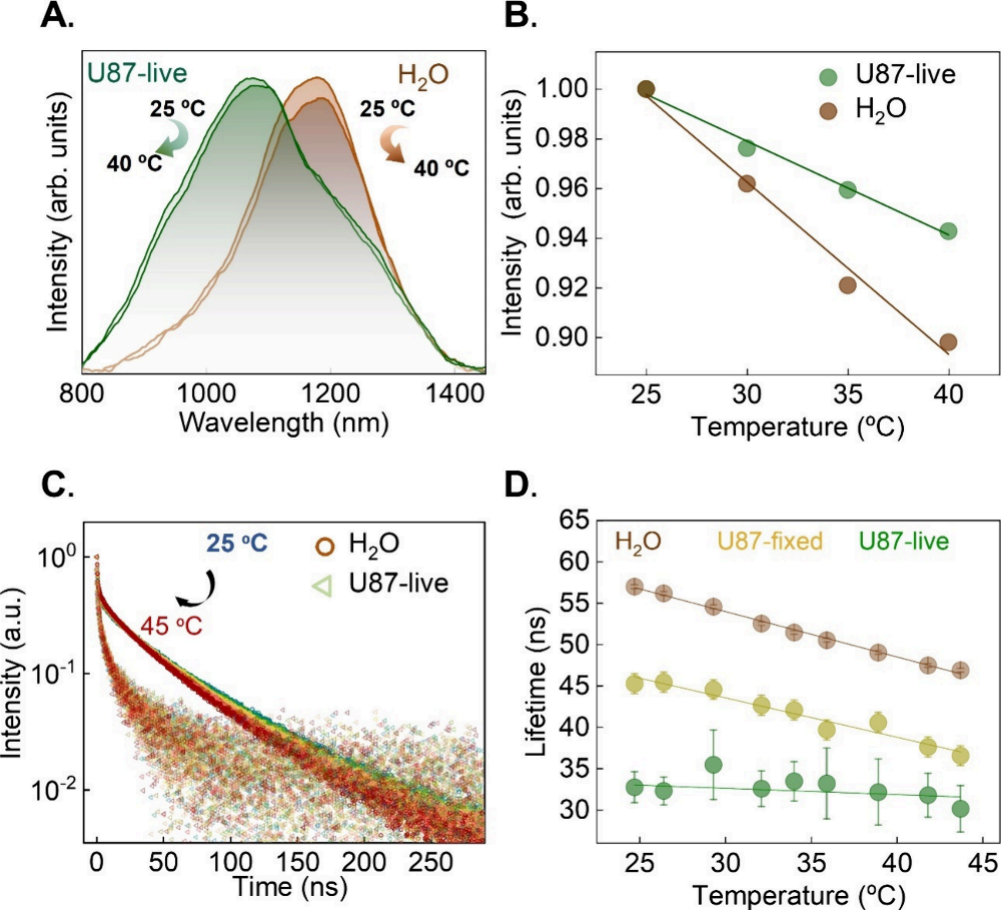
(A) Emission spectra generated by NIR-II-AuNCs at two temperatures (25 and 40 °C) as obtained when dispersed in water and within U87 live cells. (B) Temperature dependence of the emitted intensity generated by NIR-II-AuNCs dispersed in water and within U87 live cells. Symbols are experimental data, and lines are the best linear fits. (C) Luminescence decay curves corresponding to NIR-II-AuNCs in an aqueous solution and within U87 live cells, as obtained at different temperatures. (D) Temperature dependence of the average lifetime of NIR-II-AuNCs as obtained in aqueous solution and within U87 live and fixed cells. Symbols represent experimental data, and solid lines represent the best linear fits.

### Modification of the Spectroscopic Properties of NIR-II-AuNCs during In Vivo Experiments

In vitro results revealed how the spectroscopic properties of NIR-II-AuNCs are affected by interactions with a biological environment such as the cytoplasm. When NIR-II-AuNCs are used for in vivo imaging, they are exposed to interactions with different biological fluids with dynamic behavior, with the immune system and tend to accumulate in tissues and organs.^81–83^ It is therefore expected that the PL emission properties of NIR-II-AuNCs would also change during the acquisition of in vivo images. If such modifications are produced and ignored, they could induce erroneous interpretation of the in vivo fluorescence images. For instance, the acquisition of biodistribution patterns from the fluorescence images assumes a linear relation between the PL emitted intensity and the local concentration of luminescent probes. If the radiative and nonradiative rates of luminescent probes depend on the interaction with the biological media, such a linear relation cannot be assumed.

Before studying the spectroscopic features of these NIR-II-AuNCs in organs, we determine their pharmacokinetics after intravenous (i.v.) injection into the tail vein of mice (200 μL at 2 mg/mL in PBS). Based on their fluorescence signal, NIR-II-AuNCs show a half-life at 3.72 ± 0.05 min (Figure S16), which is in the range of several AuNCs.^84^ Biodistribution was assessed on U87 tumor-bearing mice (Figures S17 and S18). The results showed mainly an accumulation of NIR-II-AuNCs in the kidneys, liver, and spleen, which slightly decreases between 5 and 24 h. The in vivo and ex vivo tumor signals (Figure S19), determined between 5 and 24 h, with a tumor-to-skin and tumor-to-muscle ratios, respectively, indicate moderate accumulation and retention within the tumor. This result is expected considering the relatively fast half-life circulation in the blood, leading to rapid renal and hepatic elimination.

To explore the spectroscopic modification of NIR-II-AuNCs in in vivo environment, we used an NIR-II hyperspectral system capable of acquiring in vivo images in the 900–1700 nm range with a spectral resolution of 5 nm. NIR-II-AuNCs in PBS dispersion were intravenously administered to CD1 mice via the tail vein in a single dose of 100 μL of 4 mg/mL. The luminescence generated by NIR-II-AuNCs after 690 nm excitation was collected by an infrared optical system and focused into the hyperspectral imager (Figure 6A). The initial circulatory phase (*t*_*i*_ < 5 min, *t*_*i*_ being the time after administration of NIR-II-AuNCs) shows the NIR-II-AuNCs in the microvessels at different magnifications (Figure 6B, Videos V1 and V2). For longer circulation times (*t*_*i*_ > 5 min), the NIR-II broadband fluorescence images (acquired by integrating the fluorescence signal in the 900–1700 nm range) reveal accumulation of NIR-II-AuNCs mainly in the liver (Figure 6C). The analysis of the in vivo hyperspectral images acquired for *t*_*i*_ > 5 min makes it possible to reconstruct the in vivo emission spectra of NIR-II-AuNCs (Figure 6D). This in vivo emission spectrum differs significantly from that acquired for an aqueous solution of NIR-II-AuNCs by using the same hyperspectral imaging system (Figure 6D). Differences between emission spectra acquired in aqueous suspension and in vivo conditions have also been reported for other luminescent probes operating in the NIR-II spectral domain due to the wavelength-dependent extinction coefficient of tissues.^85^ To discard this effect in our experiments, we acquired hyperspectral images of explanted organs and tissues. The broadband PL NIR-II ex vivo images revealed accumulation of NIR-II-AuNCs mainly in the liver, but also in the spleen and kidneys (Figure S20). The ex vivo emission spectra corresponding to the liver are broadband that results from the contribution of three bands centered at 970, 1100 (dominant peak), and 1250 nm (arrows in Figure 6E). The emission peak at 970 nm comes from the autofluorescence of tissues (Figure 6F), whereas the other two bands are associated with the emission of NIR-II-AuNCs. When the contribution of autofluorescence is removed, we assess the emission spectrum of NIR-II-AuNCs within the liver (Figure 6G). When compared with the emission spectra recorded in the same system for NIR-II-AuNCs in PBS (Figure 6D), we observe an overall blueshift analogous to that observed under in vitro conditions (Figure 3A). This suggests similar physicochemical modifications of NIR-II-AuNCs in organs and in the cell environment. Indeed, a detailed analysis of the emission spectra of NIR-II-AuNCs within the liver reveals the existence of two emission bands centered at 1080 and 1254 nm (Figure S21) that have been assigned to energy transfer occurring on the AuNC surface.^35,49^ Based on in vitro results (Figure 4D,F,H), we hypothesize that once NIR-II-AuNCs accumulate in organs, they undergo simultaneous aggregation, complexation with proteins, and breakage of surface ligands.

**Figure 6:**
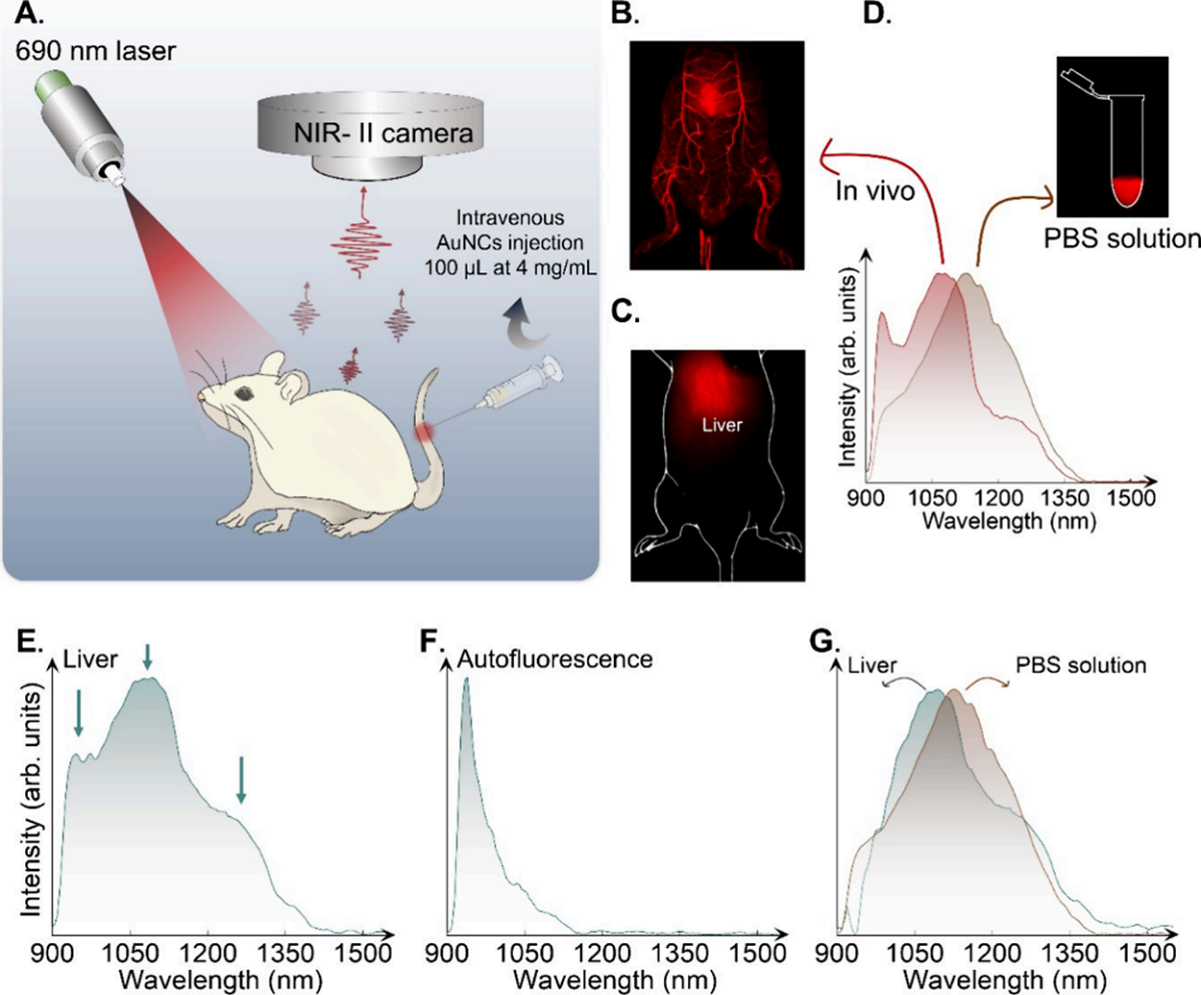
(A) Schematic representation of the experimental setup used for the acquisition of hyperspectral images and emission spectra of NIR-II-AuNCs in in vivo conditions. Broadband (900–1700 nm) whole-body fluorescence images obtained at short (*t*_*i*_ < 5 min s, (B)) and long (*t*_*i*_ > 5 min, (C)) times after intravenous injection of NIR-II-AuNCs. (D) In vivo emission spectra obtained from the analysis of hyperspectral images acquired at long times (*t*_*i*_ > 5 min) after intravenous administration of NIR-II-AuNCs. The emission spectrum obtained for a solution of NIR-II-AuNCs in PBS in the same system is also included for comparison. (E) Ex vivo emission spectra obtained from the analysis of hyperspectral images of the explanted liver. (F) Ex vivo emission spectra obtained from the analysis of hyperspectral images of skin + fat tissues associated with their autofluorescence. (G) Ex vivo emission spectra of the explanted liver after removal of autofluorescence. The emission spectra obtained from the NIR-II-AuNCs in PBS obtained in the same experimental system are also included for comparison.

### Future Design of NIR-II-AuNCs

The surface chemistry of gold nanoclusters (AuNCs) is highly sensitive to environmental factors, as demonstrated by several studies that highlight their use as fluorescent biosensors.^86–89^ In this study, we observed a significant impact of the biological environment on the fluorescence spectroscopic properties of NIR-II-AuNCs within cells and organs. These findings underscore the importance of cautious interpretation of their use in biosensing and bioimaging applications.

Various strategies exist to mitigate fluorescence spectral changes caused by interactions of AuNCs with environmental factors such as pH, reducing agents, metal complexation, and ligand exchange. For instance, enhancing the strength of ligand binding to the gold core using bidentate thiol ligands, such as derivatives of lipoic acid molecules, has been shown to improve stability.^90,91^ Similarly, stronger ligand interactions, such as those formed with N-heterocyclic carbenes (NHCs), have demonstrated promise in stabilizing AuNCs.^92,93^

To prevent aggregation and unwanted interactions with biomolecules, researchers have developed thiolated ligands with antifouling properties. These ligands are designed with tunable hydrophobicities, zwitterionic characteristics, or PEGylation, offering enhanced biocompatibility.^94–97^

Another effective strategy involves increasing ligand density through layer-by-layer techniques^98,99^ or supramolecular assembly.^100^ These approaches can reduce kernel vibrations within the gold core and limit water molecule access to the gold surface—two factors known to enhance nonradiative energy transfer.^91^

Finally, embedding AuNCs in matrices such as perovskites,^101^ silica,^102^ or polymers^34^ can significantly improve their chemical and photostability in biological environments. This approach can be applied to mitigate biases in other nanosensors. Numerous studies have demonstrated that many probes alter their optical properties upon cellular incorporation, particularly in the visible and NIR-I regions.^45,56,80,103–109^ However, the mechanisms responsible for these changes remain unclear. For NIR-II luminescent probes, such studies are even more scarce but have recently gained significant attention as a critical area of research in the development of new materials.^110^

## Conclusions

In summary, we have systematically investigated the luminescence properties of near-infrared emitting gold nanoclusters (NIR-II-AuNCs) in the second biological window within different biological environments, living cells, and organs, highlighting the sensitivity of these nanomaterials to their surroundings. Our results demonstrate that the luminescent behavior of NIR-II-AuNCs is significantly influenced by the interaction with the intracellular environment. We found that variations in the physicochemical properties of the cytoplasm (pH, ionic strength, and viscosity) have a minimum effect on the luminescence behavior of NIR-II-AuNCs. Instead, we concluded that the modification of the luminescent properties of NIR-II-AuNCs within live cells is primarily driven by the disruption of surface ligands due to the reducing conditions within the cytoplasm, alongside aggregation and complexation with biological compounds present in cells. Experiments performed in different cell lines suggested that differences in the metabolism of the cells provoked modifications in the luminescence properties of NIR-II-AuNCs. Hyperspectral in vivo experiments in murine models confirmed that the spectroscopic changes observed in vitro also manifest during in vivo applications, suggesting similar physicochemical modifications.

These findings underscore the need for a comprehensive understanding of the environmental interactions of NIR-II-AuNCs to ensure their reliable application in bioimaging and biosensing. The pronounced variability in the luminescence properties, depending on the biological context, subtracts the reliability of their use as optical sensors. As an example, we demonstrate how the modifications induced during the incorporation of NIR-II-AuNCs within live cells avoid their use as reliable thermal sensors. Therefore, future efforts should focus on engineering more stable NIR-II-AuNCs that maintain consistent luminescence properties across various biological environments. Our work provides critical insights into the development and optimization of efficient NIR-II-AuNCs, paving the way for their effective use in both in vitro and in vivo imaging and diagnostics, which represent their ultimate biological scenario.

## Experimental Methods

### Synthesis of 16 μmol NaBH4 AuMHA/HDT NCs

The gold nanoclusters (AuNCs) were synthesized by wet chemistry under alkaline conditions. Briefly, 250 μL of HAuCl4 solution (20 mM) was added to 2.4 mL of water, followed by a slow addition of a mixture of 6-mercaptohexanoic acid (MHA, 1.25 mL, 5 mM) and hexa­(ethylene glycol) dithiol (HDT, 0.75 mL, 5 mM) under mild stirring (350 rpm), keeping a molar ratio Au:MHA:HDT = 1:1.4:0.6. 250 μL of NaOH (1 M) was then added dropwise to adjust the pH to 10. Afterward, the freshly prepared reducing agent in water, NaBH4 (16 μmol, corresponding to a molar ratio Au:NaBH4 = 1:3.2), was added dropwise and stirred for 7 h at room temperature. The reaction solution was washed several times using an Amicon centrifuge filter (Milipore) 3 kDa to remove unreacted species. Then, the pH was adjusted to 7, and the NCs were kept at room temperature in the dark.

### Characterization of AuNCs

#### High-Resolution Transmission Electron Microscopy

The sizes of the metal cores were determined by high-resolution transmission electron microscopy JEOL2010 (HR-TEM) using a monochromate microscope working at 200 kV. Prior to imaging, the AuNCs were dispersed on copper grids covered with a carbon film.

#### Polyacrylamide Gel Electrophoresis

For the polyacrylamide gel electrophoresis (PAGE) experiment, separative and staking gels were prepared with total contents of acrylamide:bis-acrylamide of 25 and 2.5%, respectively. The eluting buffer consisted of 14.4 g of glycine and 3 g of tris­(hydroxymethyl)­aminoethane, diluted in 100 mL of water and adjusted to pH 7. Samples were prepared by adding 2 μL of glycerol to 20 μL of AuNCs (4 mg/mL) before depositing them in a well (15 μL per well). The gel was run with a Mini PROTEAN Bio-Rad (Hercules, CA, USA) equipment at 150 V for 2 h.

#### MALDI-tof

The molecular weight of the AuNCs was determined by MALDI-tof in positive mode using an Autoflex Speed mass spectrometer (Bruker Daltonics) in linear positive mode. A saturated solution of α-cyano-4-hydroxycinnamic acid (HCCA) in TA30 (water/acetonitrile (70/30) with 0.1% trifluoroacetic acid) was used as the matrix. The sample was prepared by diluting the nanoclusters in water to a final concentration of 0.25–0.5 mg/mL, filtering with a ziptip (Merck Milipore), and adding the matrix at a ratio of 1:1 in water.

#### Zeta Potential

The zeta potential of the sample dispersed in water (PBS, pH 7) was measured on a Zetasizer instrument from Malvern.

#### Steady-State Luminescence

Steady-state luminescence measurements were performed using a Shamrock 193i compact spectrograph and a cooled infrared photomultiplier (Hamamatsu Photonics C9525). Excitation was achieved with a 690 nm diode laser (lasing, s.a.) and a collimator. Temperature was controlled by using an air-cooled qpod 2e from Quantum Northwest.

#### Luminescence Lifetime Measurements and Sensitivity Calculation

Lifetime measurements were conducted using a thermoelectrically cooled NIR Photomultiplier tube (Hamamatsu H10330C-45) coupled to a Time-Correlated Single Photon Counting Timeharp 260 system from PicoQuant, provided with a picosecond pulsed diode laser (EPL-640) with 634.3 nm excitation and 82.4 ps pulse width. Two 850 nm long-pass filters (FELH0850 Thorlabs) were used to remove the scattered laser contribution. The equipment is provided with a grating monochromator from Oriel Instruments (77250B-MC) to select a specific lifetime emission wavelength. Temperature was controlled using an air-cooled qpod 2e from Quantum Northwest.

Sensitivity was calculated from the linear fits in Figure 5D in the main text, defined as

$$S_{r}(\%\;{}^{\circ}C^{-1})=\frac{1}{\tau }\frac{dT}{d\tau }\times 100$$

### Cell Culture Protocols

For in vitro measurements, Uppsala 87 Malignant Glioma (U87 MG cells) were cultured in phenol red Dulbecco’s modified Eagle’s medium (DMEM) supplemented with 10% fetal bovine serum (FBS), 1% penicillin–streptomycin (P/S), and l-glucose. Cells were cultured at 37 °C with 5% CO_2_. For some specific Supporting Information, RAW 264.7 macrophage and 3T3/L1 fibroblast cell lines were cultured.

### Viability

#### MTT Assay

Cell cytotoxicity was evaluated with an MTT assay. U87 cells were seeded at 104 cells/well confluence in a 96-well plate and cultured for 24 h. AuNCs were incubated for 24 h, varying the extracellular concentration [AuNCs] = 15–240 μg/mL. Cells were washed with phosphate-buffered saline (PBS) and incubated for 2 h with DMEM (without phenol red and supplemented with 10% FBS, 1% P/S) and 10% MTT. Absorption at 555 nm was measured using a microplate reader (SpectraMax Mini, Molecular Devices) at 555 nm. The reagent was used as a background control.

#### Transmission Electron Microscopy in Cells

U87 cells were incubated with NIR-AuNCs at 100 μg/mL AuNCs for 24 h, washed, and fixed with 1% glutaraldehyde. Cells were washed with phosphate buffer, postfixed in 1% buffered osmium tetroxide (1h, RT), washed in pure water, dehydrated in an ethanol series, and embedded in Quetol resin. Ultrathin sections (70 nm) were obtained with a Leica EM UC-6 ultramicrotome at room temperature. Sections were observed with a JEOL JEM- 2100-Plus microscope operating at an accelerating voltage of 200 kV. Images were recorded with a Gatan Rio 16 camera. For HR-TEM+EDS characterizations, cell samples were observed by high-resolution transmission electron microscopy JEOL2010 (HR-TEM) using a monochromated microscope working at 200 kV and coupled with an EDS detector (EDS SDD INCAEnergy TEM 100 X-Max 65 mm2) to determine the presence of gold in cellular vesicles.

#### Fluorescence Microscopy

Cells were incubated in 35 mm dishes with 100 μg/mL AuNCs for 7, 16, and 24 h. A Nikon Eclipse Ti2–U microscope with a 20× (NA = 0.45) or 60× (NA = 0.85) objective was employed. For bright field imaging, a Hamamatsu digital camera C13440 was utilized. Near-infrared fluorescence images were collected with a C-RED2 camera (First Light, Oxford Instruments) with LED source broadband illumination and a 780 nm long-pass filter (FGL780 Thorlabs). Each image was processed using ImageJ software, as an average of 50 images stacked, with an integration time of 200 ms each.

#### Co-Localization in Lysosomes

U87 cells (20,000 cells) were incubated with AuNCs (100 μg/mL) for 24 h, with an addition of Red-LysoTracker (Thermofisher) 1 h after measurements in live cells. Control experiments were performed with cells+ LysoTracker and cells+ AuNCs.

Cellular colocalization of AuNCs was visualized using a Zeiss LSM7 Live microscope (dynascope) at a 63× objective under immersion (oil). For AuNC detection, we use a 405 nm laser and a long-pass filter at 736 nm with an APD detector, while we use 570 nm excitation and 590 nm emission on a PMT detector for the detection of the lysosomes. Fluorescence quantification and the determination of Pearson’s coefficient were performed with ImageJ software.

#### Steady-State and Luminescence Lifetime Measurements

Steady-state and luminescence measurements in cells were performed in a quartz cuvette of a 3 × 3 mm light path (HellmaAnalytics). T75 flasks of confluent cells were incubated for 24 h with 100 μg/mL AuNCs (∼ 8 × 10^6^ cells). The cells were then washed with PBS and trypsinized to generate cell pellets. For fixed cells, a 2% paraformaldehyde (PFA) solution was incubated for 17 min at room temperature.

To evaluate the nature of the interaction with the cell membrane (Figure 3E,F in the main text), a T75 flask of confluent cells (∼8 × 10^6^ cells) was collected in a cell pellet. The cells were then fixed with a 2% paraformaldehyde (PFA) solution for 17 min at room temperature. Lastly, to this prefixed cell pellet, 2 μL of AuNC aqueous solution (4 mg/mL) was added by rigorously pipetting the cell pellet.

### Physiological Conditions

#### Viscosity

Glycerol was added in different quantities to a 60 μL solution of AuNCs (1 mg/mL) to obtain final solutions of 1.33, 2.56, and 5.54 cP.

#### pH

Hydrochloric acid and sodium hydroxide were employed to acidify or basify the solution, respectively.

#### Ionic Strength

Potassium hydroxide (KOH) was employed to vary the ionic strength of the AuNCs (1 mg/mL). Final concentrations of KOH in the solution were 135, 160, and 180 mM.

#### Aggregation State

Polyethylenimine hydrochloride (PEI) was employed to aggregate AuNCs. A 60 μL solution of AuNCs (1 mg/mL) was prepared, and 2 μL of 60 mM PEI was added.

#### Interaction with Proteins

The interaction with proteins was achieved by preparing 60 μL of a highly concentrated solution of bovine serum albumin (BSA) (50 mg/mL) and adding 2 μL of AuNCs at 4 mg/mL.

#### Breakage of Ligands

Dithiothreitol (DTT) was employed to break surface ligands (breakage of HDT dimer adsorbed on AuNC surface or Au-S bonds, or both) on the AuNC surface. In a 60 μL solution of AuNCs (1 mg/mL), 2 μL of 3.1 M DTT was added (final concentration of 0.1 M DTT).

### In Vivo Protocols

Animal experiments were approved and authorized by the local ethics committee under the French Ministry of Research under the reference APAFIS #33137–2021110411585349 v2. All operative procedures related to the animals strictly conformed to the Guidelines of the French Government. U87MG cells (5.106 cells in 200 μL of PBS) were injected subcutaneously in nine female NMRI nude mice (6–8 weeks old, JANVIER, France). When tumors reached ∼300 mm^3^ in volume (5 weeks), mice were anesthetized (air/isoflurane 4% for induction and 2% thereafter) and injected intravenously via the tail vein with 200 μL of Au NCs at 2 mg/mL.

### NIR-II Imaging

NIR-II imaging was performed using a Princeton camera 640ST (900–1700 nm) coupled with a laser excitation source at λ = 808 nm (120 mW/cm^2^) and a Kaer Labs image acquisition software. We used a short-pass excitation filter at 1000 nm (Thorlabs) and long-pass filters on the NIR-II camera at 1250 or 1400 nm (Thorlabs). A 25 mm or a 50 mm lens with an N.A. = 4 aperture (Navitar) was used to focus on the samples. Analyses were performed using FIJI software.

### Injection Study

Mice were injected with AuNCs (200 μL; 2 mg/mL in PBS) in the tail vein, and images were recorded during the injection and up to 20 min afterward (exposure time: 500 ms).

### Pharmacokinetic Studies

Mice (*n* = 3) were injected for blood sample collection at 5, 10, 15, 20, 30, 45, 60 min, and 24 h after injection. Blood samples were centrifuged (700 g, 10 min, 4 °C) to separate the plasma. Ten (10) μL plasma samples were imaged by NIR-II fluorescence, and plasma pharmacokinetics were analyzed through a noncompartment model (GraphPad Prism 7.00, GraphPad Software, La Jolla California USA).

### Biodistribution Studies

Mice (*n* = 6) were anesthetized (air/isoflurane 4% for induction and 2% thereafter), and whole-body fluorescence imaging was performed before and at 1 h 30 min, 3h, 5h, and 24h after injection. At 5 and 24 h, three mice were euthanized, and the organs were harvested for ex-vivo fluorescence imaging.

### Hyperspectral Images

Animal experiments were approved and authorized by the local ethics committee under the Spanish Ministry of Research under the reference PROEX 58.7-23.

CD1 male mice were anesthetized by isofluorane inhalation (4% for induction and 2% thereafter) through a SomnoSuite Low-Flow Anesthesia System (Kent Scientific, Connecticut, USA). Mice were then injected with AuNCs (100 μL;4 mg/mL in PBS) in the tail vein. Hyperspectral images were acquired in real time in order to monitor the biodistribution of the nanoparticles. For this purpose, fluorescence images based on the 1000–1700 nm emissions were acquired by illuminating the whole body of the mouse with a 690 nm fiber-coupled diode, with an hyperspectral imaging cube.

## Supplementary Material







## Notes

AuMHAHDT 800 25mm (AVI)
AuMHAHDT 800 50mm (AVI)
Characterization of 16 μmol NaBH_4_ AuMHA/HDT NCs; MALDI-TOF pattern; PAGE electrophoresis; cytotoxicity assay; decay fitting; and fitting of decay curves (PDF)
