# Supplementary material for: Luminescence Fingerprint of Intracellular NIR-II Gold Nanocluster Transformation: Implications for Sensing and Imaging
Source: ACS Nano. 2025 Feb 24;19(8):7821–34. doi: 10.1021/acsnano.4c13955 (PMC12129262; doi:10.1021/acsnano.4c13955)
Supplement: Supplementary file 3 [file nn4c13955_si_003.pdf]

Luminescence Fingerprint of Intracellular NIR-II Gold Nanocluster Transformation: Implications for Sensing and Imaging

Marina París Ogáyar[a], Zeineb Ayed[b], Veronique Josserand[b], Maxime Henry[b], Álvaro Artiga[a], Livia Didonè[c], Miriam Granado[c], Aida Serrano[d], Ana Espinosa[e], Xavier Le Guével[b] and Daniel Jaque[a,f*]*

[a] Nanomaterials for BioImaging Group (nanoBIG), Facultad de Ciencias, Departamento de Física de Materiales, Universidad Autónoma de Madrid, 28049 Madrid, Spain.
[b] University Grenoble Alpes, INSERM U1209, CNRS UMR5309, Institute for Advanced Biosciences F-38000 Grenoble, France
[c] Nanomaterials for BioImaging Group (nanoBIG), Facultad de Medicina, Departamento de Fisiología, Universidad Autónoma de Madrid, 28029 Madrid, Spain.
[d] Instituto de Cerámica y Vidrio| CSIC. Campus de Cantoblanco, 28049 Madrid, Spain.
[e] Instituto de Ciencia de Materiales de Madrid | CSIC. Campus de Cantoblanco, 28049 Madrid, Spain.
[f] Institute for Advanced Research in Chemical Sciences (IAdChem), Universidad Autónoma de Madrid, Madrid 28049, Spain.

Table of contents

Results

(**S1**) Characterization of 16 µmol $NaBH_4$ AuMHA/HDT NCs

(**S2**) MALDI-TOF pattern

(**S3**) PAGE electrophoresis

(**S4**) Cytotoxicity assay and fluorescence microscopy of AuNCs in U87-MG cell line

(**S5**) Endosomal uptake: Energy-Dispersive X-ray Spectroscopy and confocal fluorescence microscopy

(**S6**) Extinction coefficient of a cell pellet

(**S7**) Decay fitting of NIR-II-AuNCs in water and within cells

(**S8**) Fluorescence decay curves obtained in solutions with different pH

(**S9**) Effect of basic pH

(**S10**) Fluorescence decay curves obtained in solutions with different viscosity

(**S11**) Fluorescence decay curves obtained in solutions with different ionic strength

(**S12**) Evidence of AuNC aggregation induced by polyethyleneimine (PEI)

(**S13**) Fluorescence microscopy of AuNCs in RAW 264.7 macrophage and 3T3/L1 fibroblasts cell lines

(**S14**) Spectroscopic properties of AuNCs in RAW 264.7 macrophage and 3T3/L1 fibroblasts cell lines

(**S15**) Calibration curves and sensitivity

(S16) Calibration in plasma and pharmacokinetic

(**S17-S19**) In-vivo and ex-vivo fluorescence imaging

(**S20**) Broadband photoluminescence NIR-II ex vivo images of different organs

(**S21**) Deconvolution of the ex vivo emission spectra of explanted liver

(Table S1) Fitting of decay curves at 25 ºC in different media or under different conditions.

(S1) Characterization of 16 µmol $NaBH_4$ AuMHA/HDT NCs

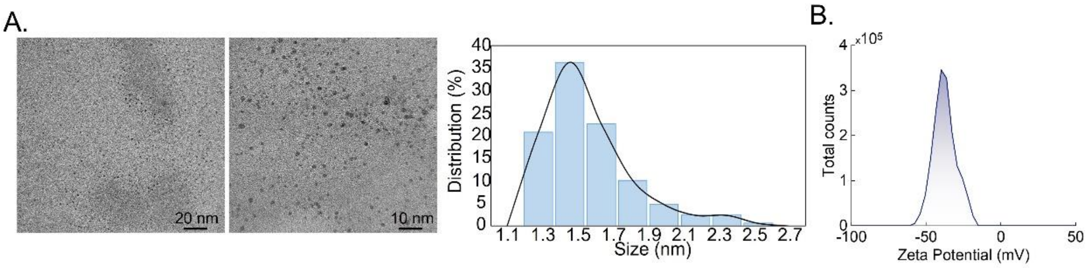

Figure S1. A) Transmission electron microscopy (TEM) images and size distribution (200 particles) of AuNCs. B) Z Potential at pH 7.

(S2) MALDI-TOF pattern

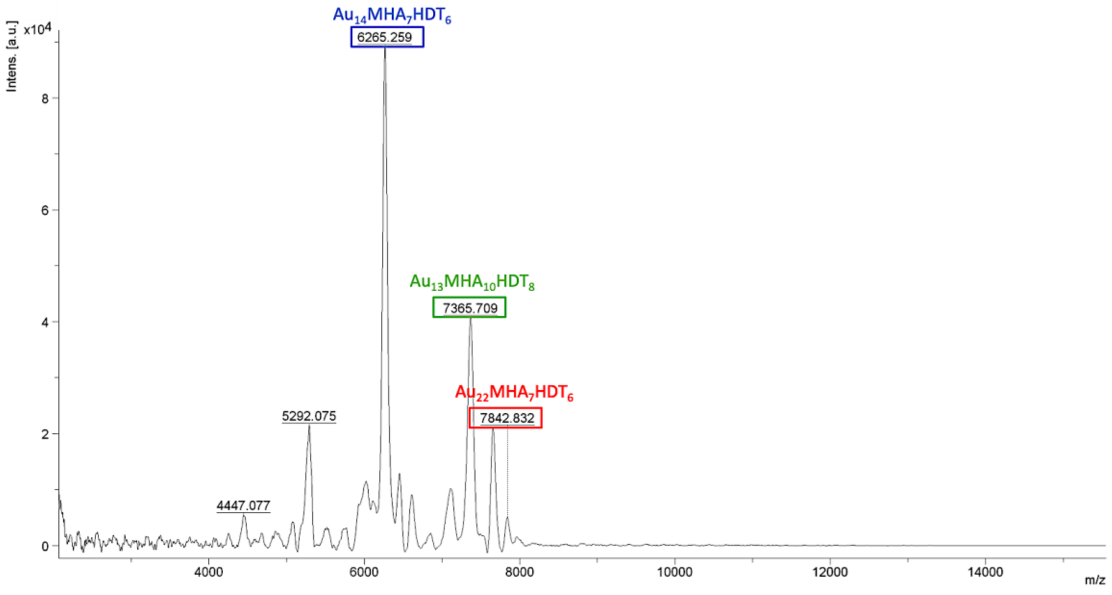

Figure S2. Matrix assisted laser desorption/ionization time-of-flight mass spectrometry (MALDI-TOF) pattern of AuNCs recorded in positive mode.

(S3) PAGE electrophoresis

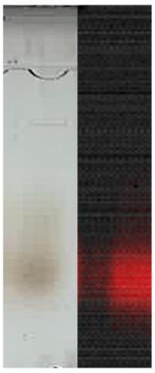

Figure S3. PAGE electrophoresis of AuNCs sample. ($\lambda_{exc.}$= 808 nm; $\lambda_{em.}$= 1064-1700 nm).

(S4) Cytotoxicity assay and fluorescence microscopy of AuNCs in U87-MG cell line

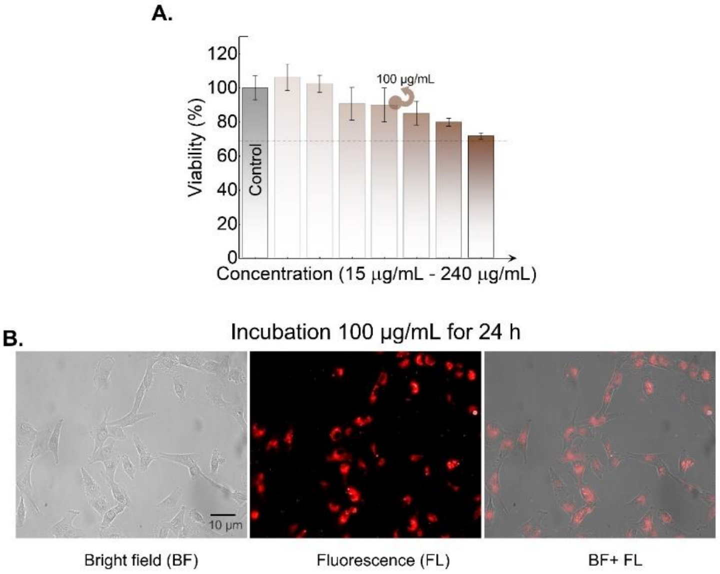

Figure S4. Cellular uptake and biocompatibility of AuNCs in U87 cells. A) Cell viability assessed by MTT assay after 24 h incubation with varying AuNCs extracellular concentrations (15-240 μg/mL) at 37 °C. Solid line indicates the standardized guideline

for toxicity (70%). B) Fluorescence microscopy images with selected conditions (AuNC concentration of 100 µg/mL and 24 h incubation time) for subsequent studies.

(S5) Endosomal uptake: Energy-Dispersive X-ray Spectroscopy and confocal fluorescence microscopy

AuNCs typically have a core size smaller than 3 nm and a hydrodynamic diameter of less than 10 nm. Several studies have demonstrated that non-targeted zwitterionic and pegylated-protected gold nanoclusters accumulate on the cell surface before being internalized via endocytic pathways, primarily involving clathrinid-mediated pathways and micropinocytosis, among others. [1-4] For example, zwitterionic-protected AuNCs are sequestered in lysosomal vacuoles within fibroblasts.[5] Cellular uptake, in both normal and cancer cells, is concentration and time-dependent, with high variability depending on the cell type and the nature of the ligands protecting the gold nanoclusters.[6-8]

Modifying the surface properties of AuNCs—for instance, by introducing positive charges or increasing hydrophobicity—can enhance cellular uptake[9, 10] and impact organelle morphology, such as mitochondria[5]. However, the detailed mechanisms underlying these uptake pathways remain underexplored.

In our study, AuMHA/HDT nanoclusters exhibit a slightly negative surface charge due to the carboxyl groups of the MHA ligand and possess pegylated properties derived from the HDT ligand. Previous reports on cellular uptake in various cell lines (lung cancer A549, breast cancer 4T1, HEK)[11] and in this work (U87 glioblastoma, RAW 264.7 macrophages, and 3T3/L1 fibroblasts) indicate behavior similar to pegylated and zwitterionic-protected AuNCs. Notably, the uptake pathway appears comparable to that of smaller gold nanoparticles (5–20 nm) with similar surface chemistry.[12, 13]

Specific ligands introduced on the AuNC surface—either during synthesis or via ligand exchange—have been reported to target receptors on the cell surface, such as integrins[14], as well as intracellular targets like mitochondria[15] or the nucleus[16]. Strategies to enable endosomal escape, such as incorporating cell-penetrating peptides on the AuNC surface, have also been explored.[17]

Transmission Electron Microscopy (TEM) with Energy-Dispersive X-ray Spectroscopy (EDX/EDS): Additionally, we performed TEM analysis coupled with EDX/EDS to confirm the elemental presence of gold (Au) within the endosomal compartments. This analysis

corroborates the fluorescence microscopy findings, further validating the localization of the NIR-II-AuNCs within the endosomes.

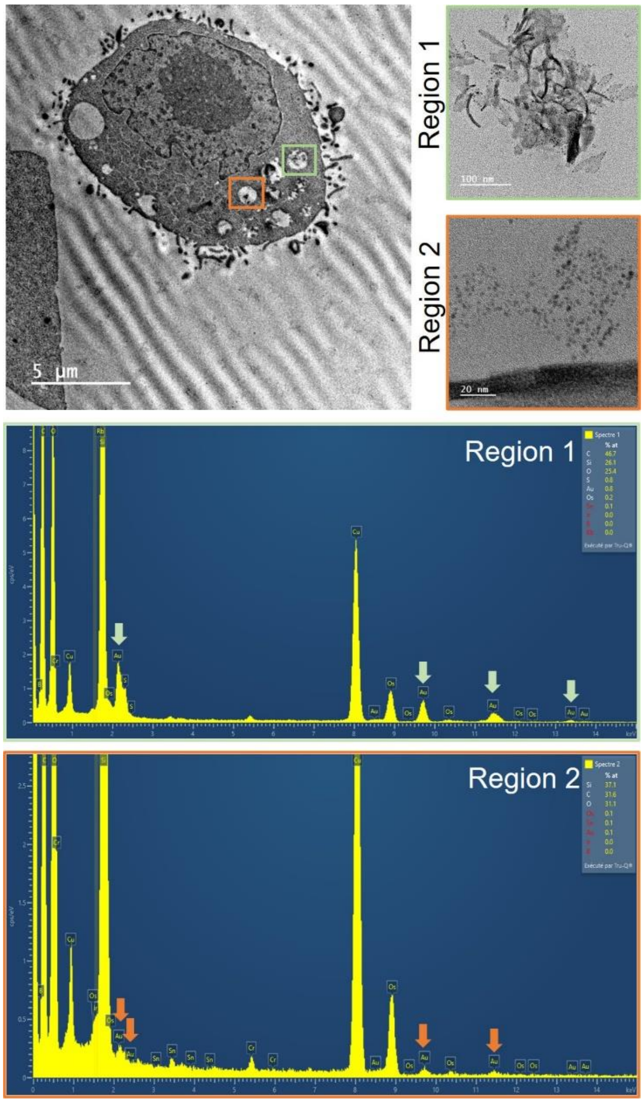

Figure S5A. HRTEM and EDX measurements on U87 incubated with NIR-II-AuNCS at 100 µg/mL for 24 hours showing the presence of ultra-small gold particles in intracellular vesicles. Arrows indicate the signal corresponding to Au.

Confocal Fluorescence Microscopy: We utilized confocal fluorescence microscopy combined with specific staining of lysosomes. The results demonstrate a clear co-localization of the endosome markers with the fluorescence signal of the NIR-II-AuNCs, providing evidence of their presence in the late endosomes with a Pearson coefficient above 0.5 (r= 0.67) for the colocalization between lysosomes and the NIR-II-AuNCs.

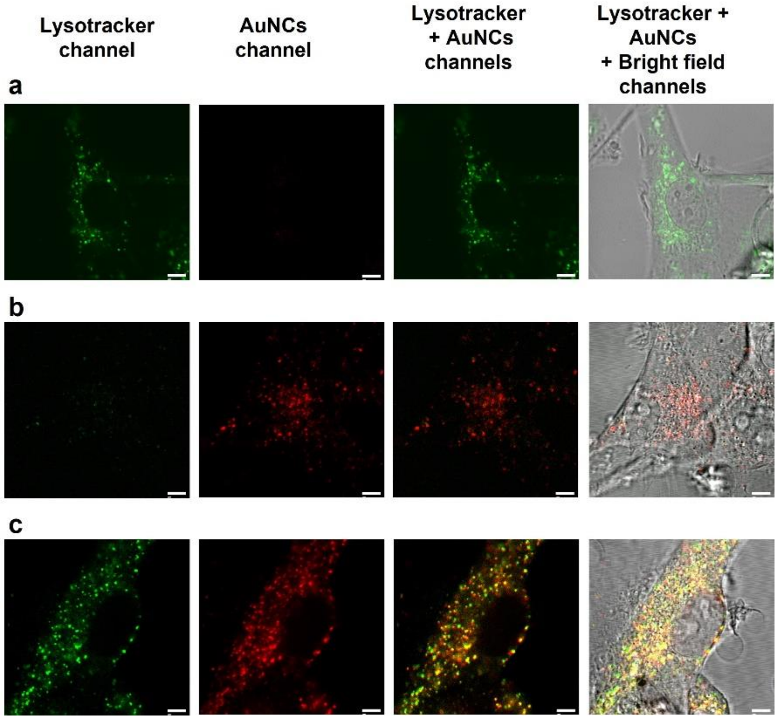

Figure S5B. Confocal fluorescence microscopy measurements experiment of NIR-II-AuNCs co-localization within U87 cell line lysosomes. Control experiments were performed with **a)** incubation of lysotracker without AuNCs, **b)** incubation of AuNCs without lysotracker. Co-localization experiment of lysotracker + AuNCs is shown in part **c)**. For b) and c) 100 µg/mL of NIR-II-AuNCs were incubated for 24h. Scale bars : 5µM.

(S6) Extinction coefficient of a cell pellet

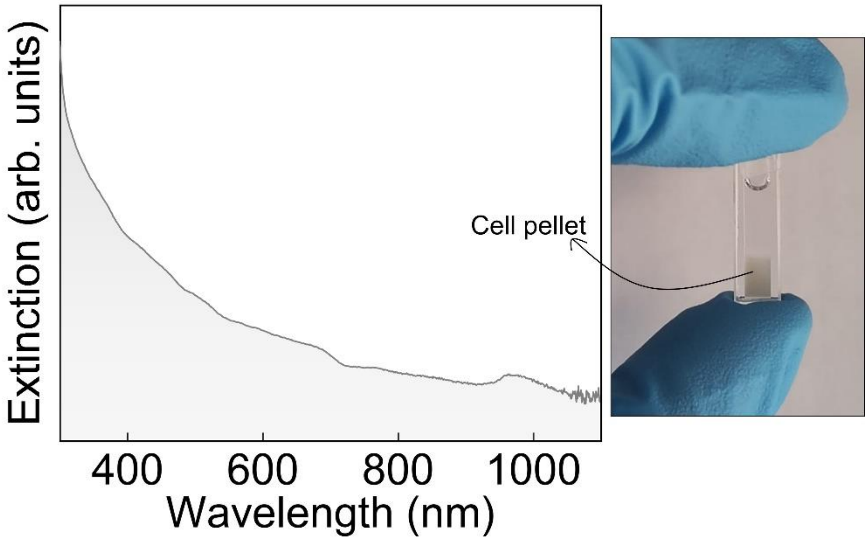

Figure S6. Extinction coefficient of a cell pellet.

(S7) Decay fitting of NIR-II-AuNCs in water and within cells

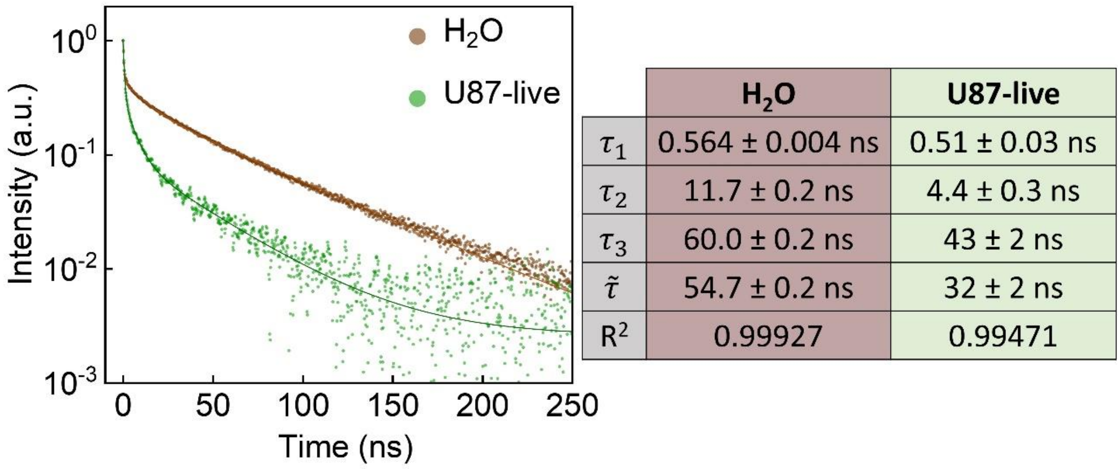

	H_2O	U87-live
τ_1	0.564 ± 0.004 ns	0.51 ± 0.03 ns
τ_2	11.7 ± 0.2 ns	4.4 ± 0.3 ns
τ_3	60.0 ± 0.2 ns	43 ± 2 ns
$\tilde{\tau}$	54.7 ± 0.2 ns	32 ± 2 ns
R^2	0.99927	0.99471

Figure S7. Decay fitting of NIR-II-AuNCs in water and within cells.

(S8) Fluorescence decay curves obtained in solutions with different pH

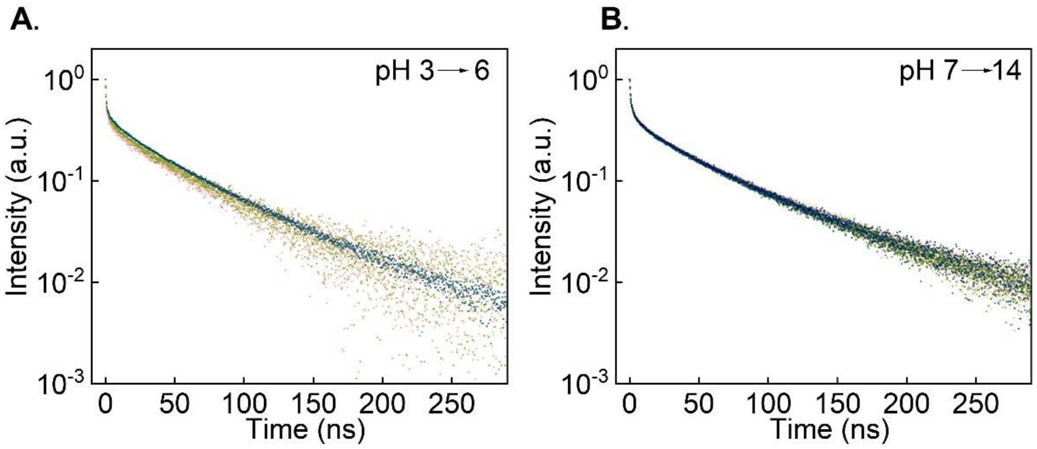

Figure S8. Fluorescence decay curves obtained in solutions with different pH. A) Datasets for Figure 4B in main text. B) Datasets for Figure S8 in the supporting information.

(S9) Effect of basic pH

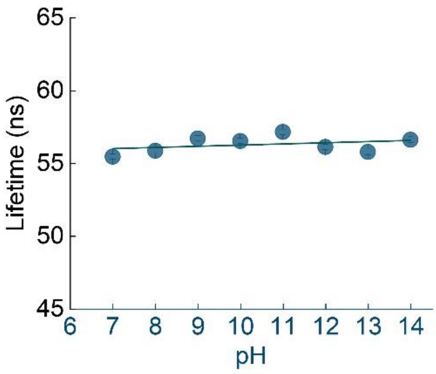

Figure S9. Average PL lifetime decay at 25 °C as a function of basic pH (range 7.0 to 14.0).

(S10) Fluorescence decay curves obtained in solutions with different viscosity

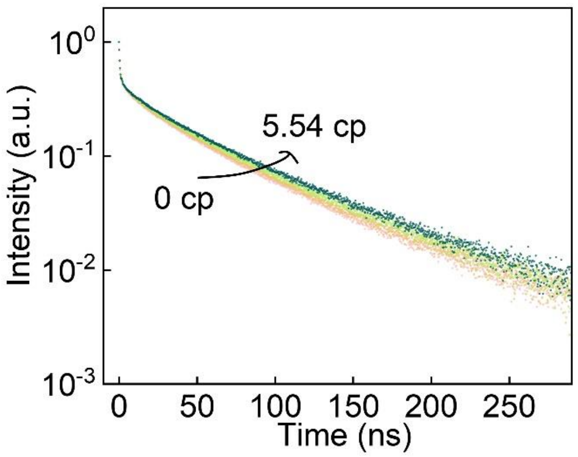

Figure S10. Fluorescence decay curves obtained in solutions with different viscosity. Datasets for Figure 4B in main text.

(S11) Fluorescence decay curves obtained in solutions with different ionic strength

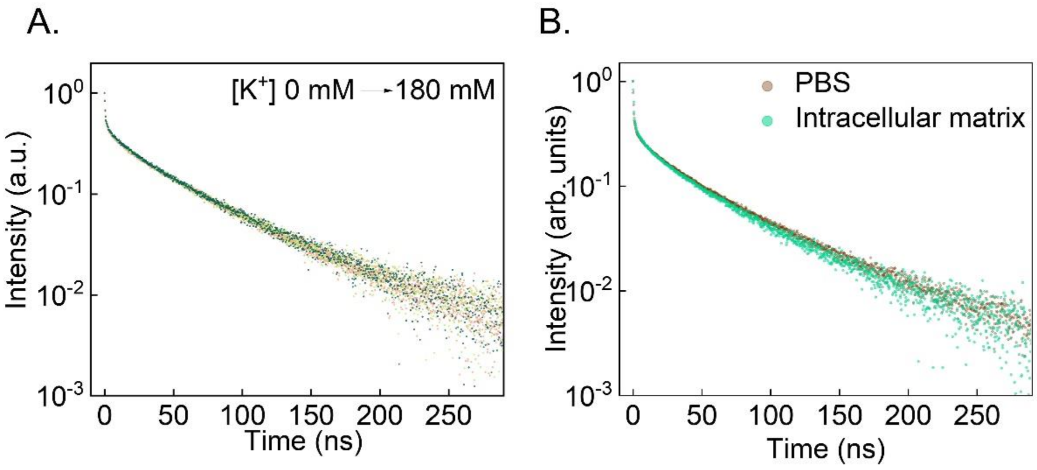

Figure S11. Fluorescence decay curves obtained **A)** in aqueous solutions with different ionic strength (datasets for Figure 4B in main text) and **B)** in PBS and within an intracellular matrix composed of PBS and ions (Na^{+}, K^{+} and Fe^{3+} at 15, 150 and 3 mM, respectively). [18-21]

(S12) Evidence of AuNC aggregation induced by polyethyleneimine (PEI)

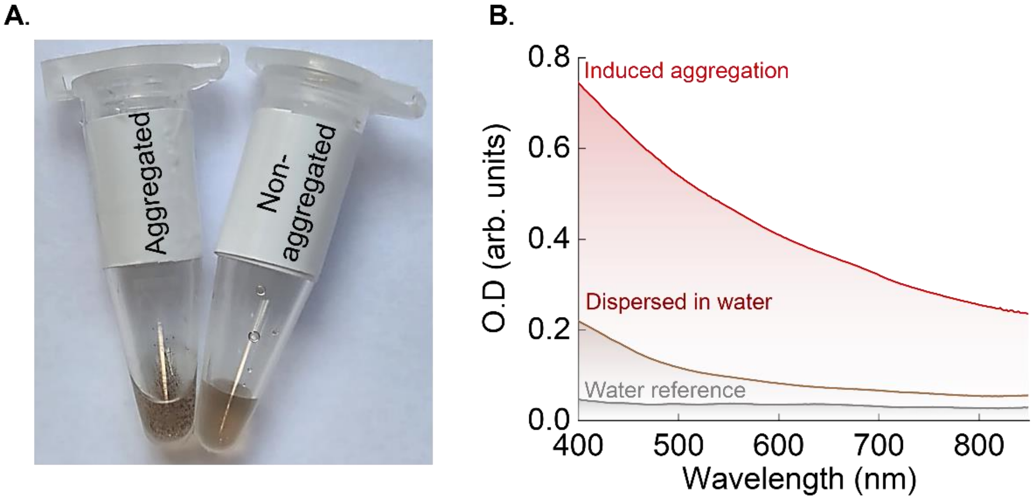

Figure S12. Evidence of AuNC aggregation induced by polyethyleneimine (PEI). (A) Images taken for more than 24 h after inducing aggregation with PEI. The aggregated sample shows obvious turbidity. (B) Optical density spectra of the AuNCs before and after inducing aggregation. Light scattering/absorption increases with the aggregation of AuNCs.

(S13) Fluorescence microscopy of AuNCs in RAW 264.7 macrophage and 3T3/L1 fibroblasts cell lines

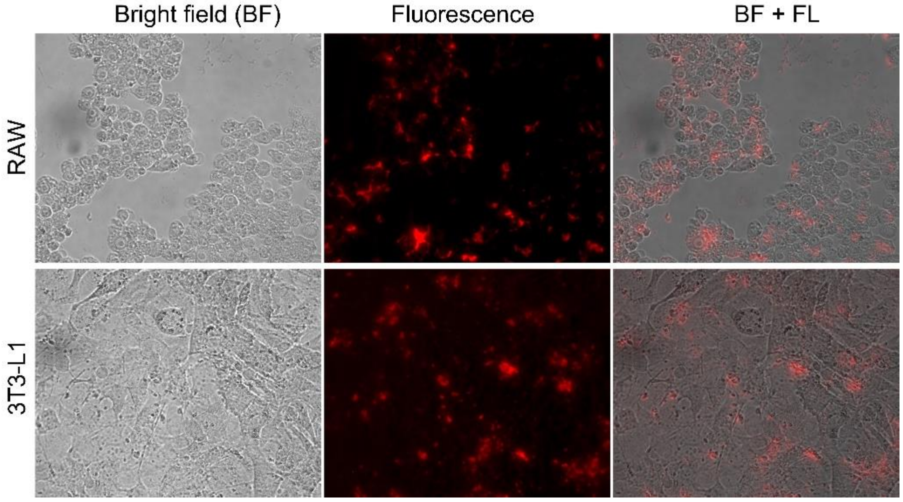

Figure S13. Fluorescence microscopy images with selected conditions (AuNC concentration of 100 µg/mL and 24 h incubation time) in RAW 264.7 macrophage and 3T3/L1 fibroblasts cell lines.

(S14) Spectroscopic properties of AuNCs in RAW 264.7 macrophage and 3T3/L1 fibroblasts cell lines

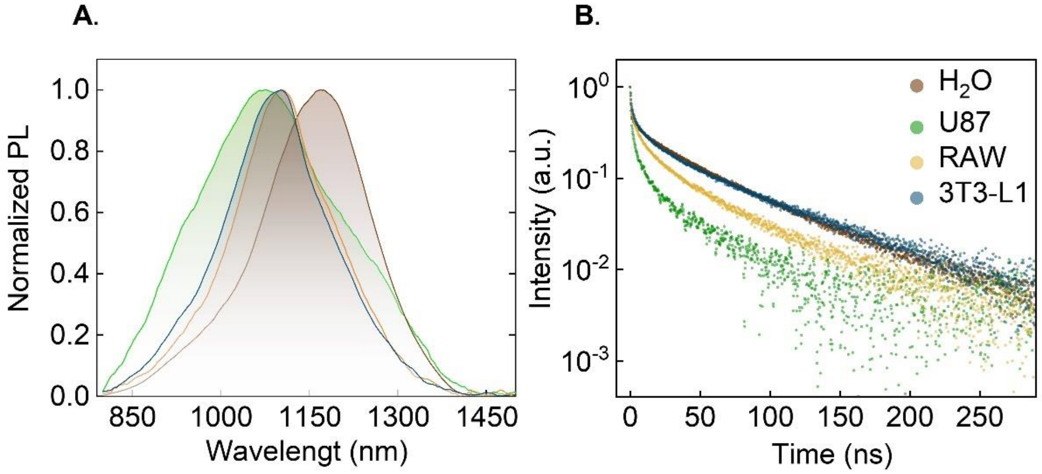

Figure S14. Comparison of the emission spectra **(A)** and decay curves **(B)** corresponding to NIR-II-AuNCs dispersed in water and within U87, RAW 264.7 macrophage and 3T3/L1 fibroblasts cell lines.

(S15) Calibration curves and sensitivity

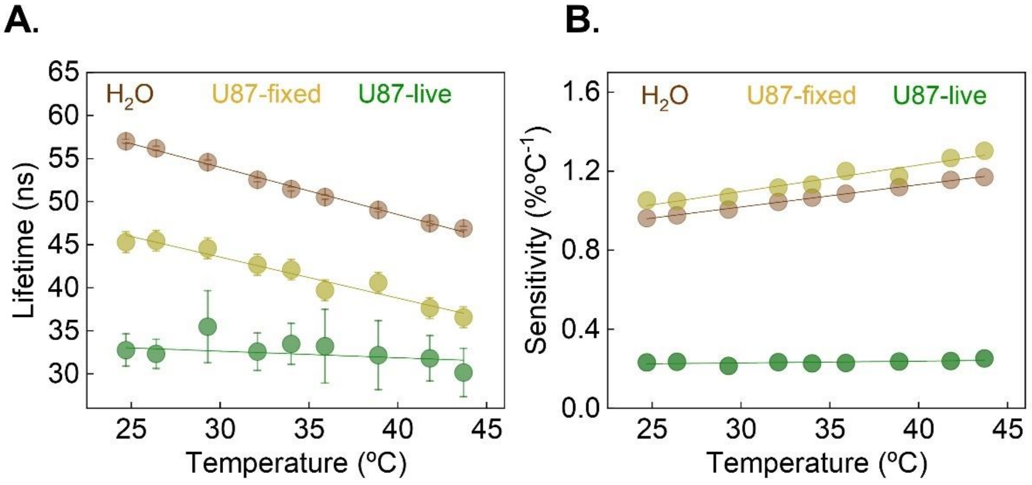

Figure S15. Calibration curves of **(A)** average lifetime and **(B)** sensitivity as a function of temperature of AuNCs in water and within U87-live and U87-fixed.

(S16) Calibration in plasma and pharmacokinetic

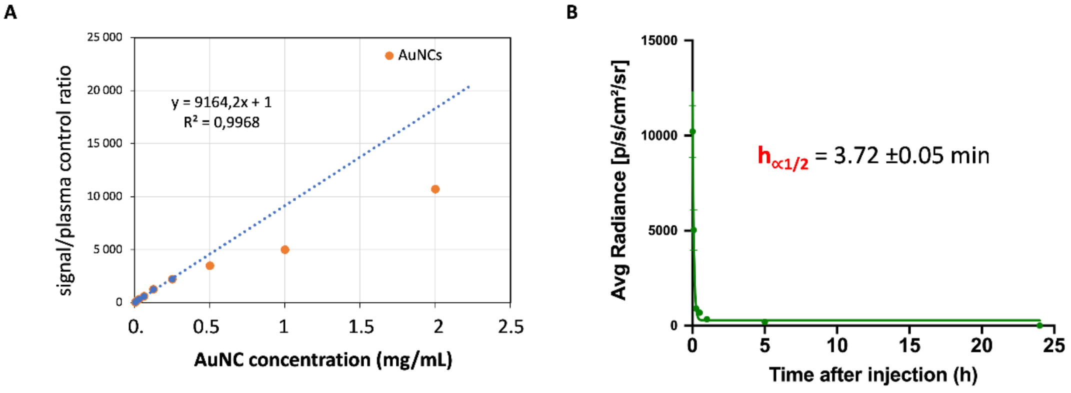

Figure S16. **A)** Calibration of AuNCs diluted in plasma from 10mg/mL to 2 mg/mL and measured by NIR-II imaging. ($\lambda_{exc.}$= 808 nm; $\lambda_{em.}$= 1064-1700 nm). **B**) Pharmacokinetic of AuNCs after injection in mice (200µL at 2 mg/mL). Mean ±SEM N= 3 mice.

(S17-S19) In-vivo and ex-vivo fluorescence imaging

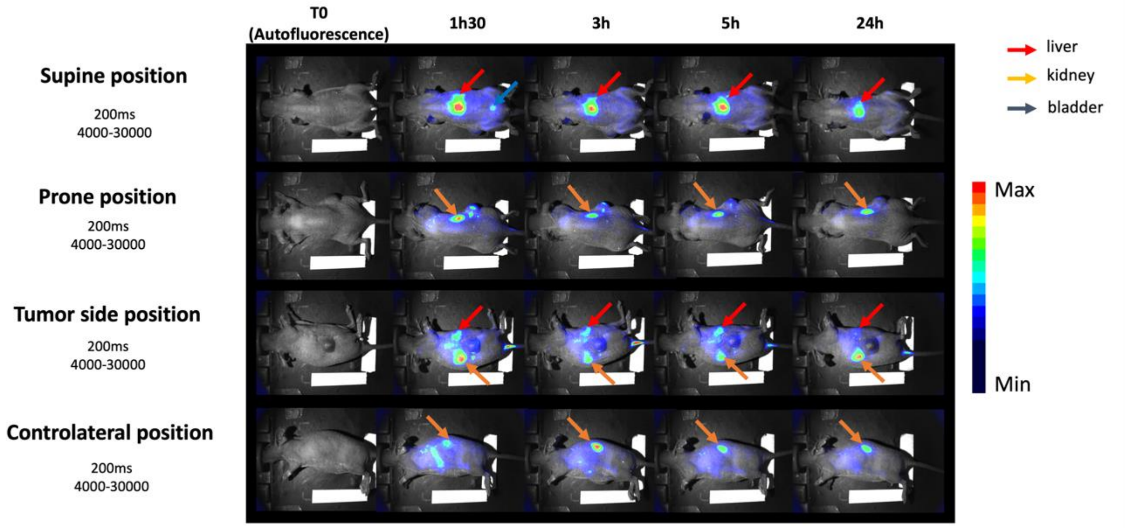

Figure S17. *In vivo* whole-body fluorescence imaging after intravenous injection of AuNCs in subcutaneous tumor bearing mice. Data from a mouse representative of the group: s17.

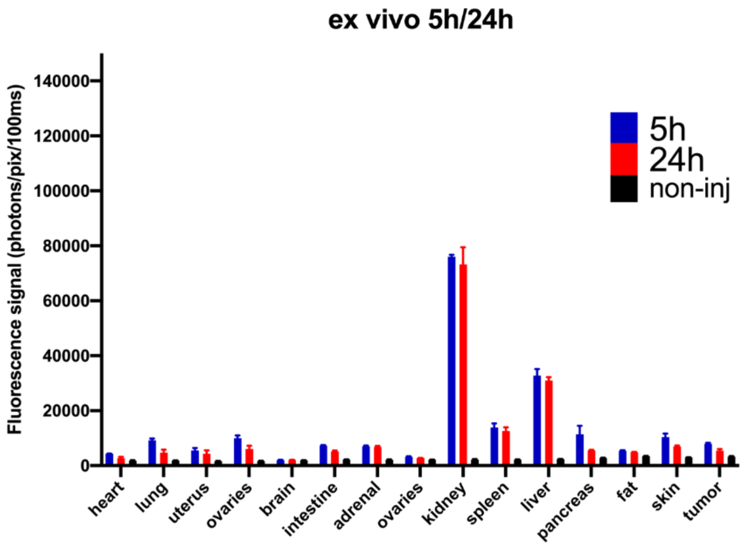

Figure S18. *Ex vivo* fluorescence imaging on isolated tissues 5 h and 24 h after intravenous injection in subcutaneous tumor bearing mice. Mean±SEM (n=3 at 5 h, n=3 at 24 h, n=2 CTL). CTL: autofluorescence from non-injected mice.

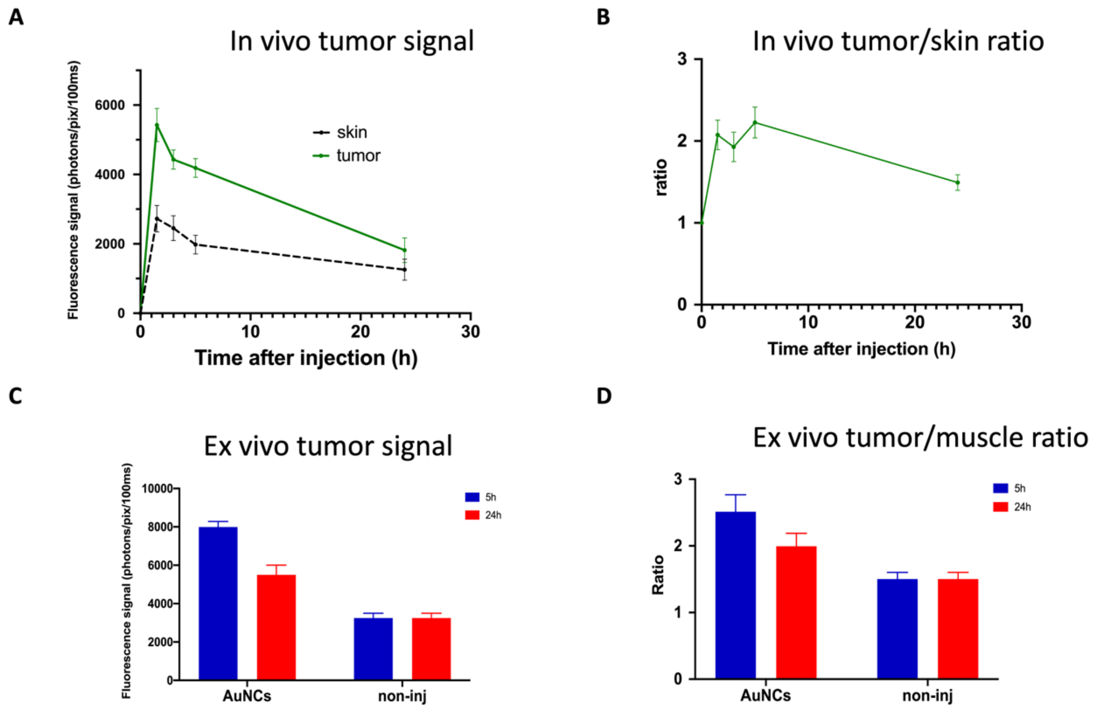

Figure S19. **A, B**. *In vivo* whole-body fluorescence imaging after intravenous injection in subcutaneous tumor bearing mice. **C, D**. *Ex vivo* fluorescence imaging on isolated tissues 5 h and 24 h after intravenous injection in subcutaneous tumor bearing mice. Mean±SEM (n=3 at 5 h, n=3 at 24 h, n=2 CTL). CTL: autofluorescence from non-injected mice.

(S20) Broadband photoluminescence NIR-II ex vivo images of different organs

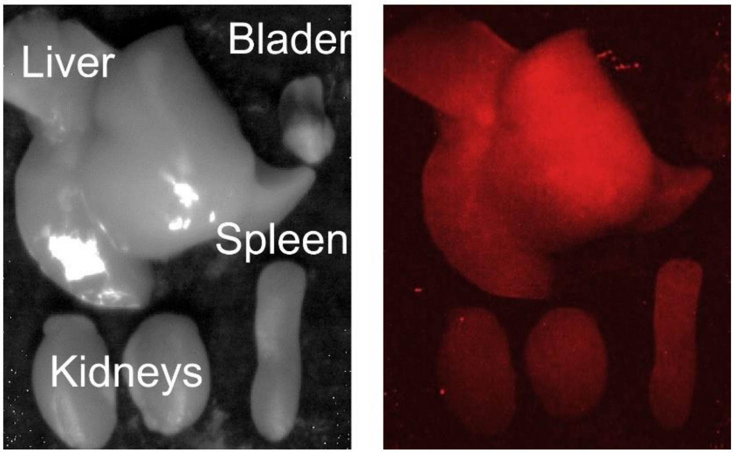

Figure S20. Broadband photoluminescence NIR-II *ex vivo* images of different organs.

(S21) Deconvolution of the ex vivo emission spectra of explanted liver

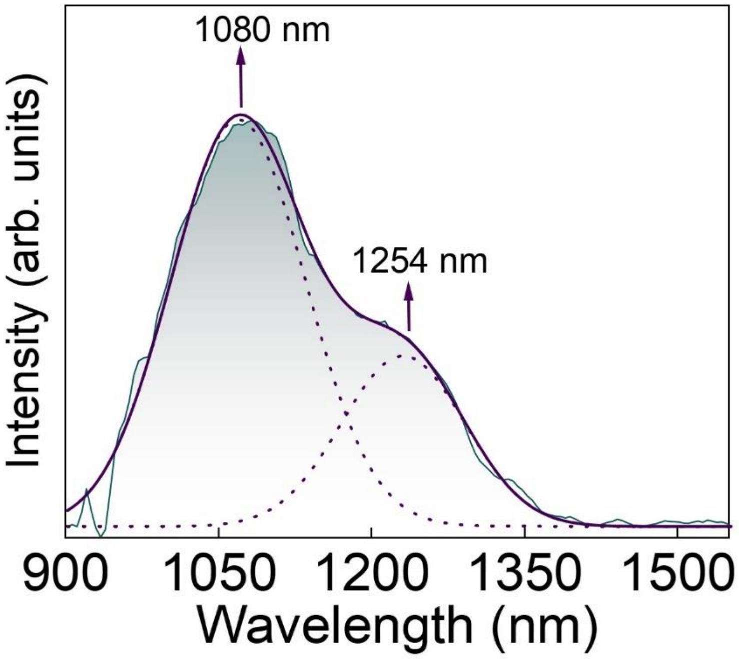

Figure S21. Deconvolution of the ex vivo emission spectra of explanted liver.

	**H_2O**	**U87-live**	**DMEM**	**Membrane**	**Aggregated**	**BSA**	**DTT**
A_1	0.547 ± 0.002	0.60 ± 0.02	0.558 ± 0.004	0.639 ± 0.004	0.78 ± 0.01	0.611 ± 0.005	0.825 ± 0.004
τ_1	**0.564 ± 0.004 ns**	**0.51 ± 0.03 ns**	**0.581 ± 0.009 ns**	**0.509 ± 0.006 ns**	**0.60 ± 0.02 ns**	**0.82 ± 0.01 ns**	**0.621 ± 0.006 ns**
A_2	0.160 ± 0.001	0.30 ± 0.01	0.160 ± 0.003	0.159 ± 0.002	0.082 ± 0.006	0.152 ± 0.005	0.139 ± 0.003
τ_2	**11.7 ± 0.2 ns**	**4.4 ± 0.3 ns**	**11.9 ± 0.4 ns**	**8.8 ± 0.2 ns**	**8 ± 1 ns**	**16.9 ± 0.8 ns**	**6.9 ± 0.3 ns**
A_3	0.300 ± 0.001	0.103 ± 0.004	0.284 ± 0.003	0.197 ± 0.002	0.134 ± 0.004	0.288 ± 0.006	0.061 ± 0.002
τ_3	**60.0 ± 0.2 ns**	**43 ± 2 ns**	**60.5 ± 0.5 ns**	**60.1 ± 0.5 ns**	**62 ± 1 ns**	**69.5 ± 0.9 ns**	**56 ± 1 ns**
$\bar{\tau}$	**54.7 ± 0.2 ns**	**32 ± 2 ns**	**54.8 ± 0.5 ns**	**53.4 ± 0.2 ns**	**55 ± 1 ns**	**62.1 ± 0.9 ns**	**40.8 ± 1 ns**
R^2	0.99927	0.99471	0.997	0.99576	0.96186	0.99537	0.99011

Table S1. Fitting of decay curves at 25 ºC in different media or under different conditions.

References

1. Le Guével, X.; Perez Perrino, M.; Fernández, T. D.; Palomares, F.; Torres, M. J.; Blanca, M.; Rojo, J.; Mayorga, C., Multivalent Glycosylation of Fluorescent Gold Nanoclusters Promotes Increased Human Dendritic Cell Targeting via Multiple Endocytic Pathways. *ACS Appl Mater Interfaces* **2015,** *7* (37), 20945-56.
2. Yang, L.; Shang, L.; Nienhaus, G. U., Mechanistic aspects of fluorescent gold nanocluster internalization by live HeLa cells. *Nanoscale* **2013,** *5* (4), 1537-1543.
3. Sang, D.; Luo, X.; Liu, J., Biological Interaction and Imaging of Ultrasmall Gold Nanoparticles. *Nanomicro Lett* **2023,** *16* (1), 44.
4. Sokolova, V.; Ebel, J. F.; Kollenda, S.; Klein, K.; Kruse, B.; Veltkamp, C.; Lange, C. M.; Westendorf, A. M.; Epple, M., Uptake of Functional Ultrasmall Gold Nanoparticles in 3D Gut Cell Models. *Small* **2022,** *18* (31), e2201167.
5. Linklater, D. P.; Le Guével, X.; Kosyer, E.; Rubanov, S.; Bryant, G.; Hanssen, E.; Baulin, V. A.; Pereiro, E.; Perera, P. G. T.; Wandiyanto, J. V.; Angulo, A.; Juodkazis, S.; Ivanova, E. P., Functionalized Gold Nanoclusters Promote Stress Response in COS-7 Cells. *Advanced NanoBiomed Research* **2023,** *3* (4), 2200102.
6. Fernández, T. D.; Pearson, J. R.; Leal, M. P.; Torres, M. J.; Blanca, M.; Mayorga, C.; Le Guével, X., Intracellular accumulation and immunological properties of fluorescent gold nanoclusters in human dendritic cells. *Biomaterials* **2015,** *43*, 1-12.
7. Zhang, C.; Zhou, Z.; Zhi, X.; Ma, Y.; Wang, K.; Wang, Y.; Zhang, Y.; Fu, H.; Jin, W.; Pan, F.; Cui, D., Insights into the distinguishing stress-induced cytotoxicity of chiral gold nanoclusters and the relationship with GSTP1. *Theranostics* **2015,** *5* (2), 134-49.
8. Tay, C. Y.; Yu, Y.; Setyawati, M. I.; Xie, J.; Leong, D. T., Presentation matters: Identity of gold nanocluster capping agent governs intracellular uptake and cell metabolism. *Nano Research* **2014,** *7* (6), 805-815.
9. Porret, E.; Fleury, J. B.; Sancey, L.; Pezet, M.; Coll, J. L.; Le Guével, X., Augmented interaction of multivalent arginine coated gold nanoclusters with lipid membranes and cells. *RSC Adv* **2020,** *10* (11), 6436-6443.
10. Porret, E.; Sancey, L.; Martín-Serrano, A.; Montañez, M. I.; Seeman, R.; Yahia-Ammar, A.; Okuno, H.; Gomez, F.; Ariza, A.; Hildebrandt, N.; Fleury, J.-B.; Coll, J.-L.; Le Guével, X., Hydrophobicity of Gold Nanoclusters Influences Their Interactions with Biological Barriers. *Chemistry of Materials* **2017,** *29* (17), 7497-7506.
11. Yu, Z.; Musnier, B.; Wegner, K. D.; Henry, M.; Chovelon, B.; Desroches-Castan, A.; Fertin, A.; Resch-Genger, U.; Bailly, S.; Coll, J.-L.; Usson, Y.; Josserand, V.; Le Guével, X., High-Resolution Shortwave Infrared Imaging of Vascular Disorders Using Gold Nanoclusters. *ACS Nano* **2020,** *14* (4), 4973-4981.
12. Ho, L. W. C.; Yung, W. Y.; Sy, K. H. S.; Li, H. Y.; Choi, C. K. K.; Leung, K. C.; Lee, T. W. Y.; Choi, C. H. J., Effect of Alkylation on the Cellular Uptake of Polyethylene Glycol-Coated Gold Nanoparticles. *ACS Nano* **2017,** *11* (6), 6085-6101.
13. Jiang, Y.; Huo, S.; Mizuhara, T.; Das, R.; Lee, Y. W.; Hou, S.; Moyano, D. F.; Duncan, B.; Liang, X. J.; Rotello, V. M., The Interplay of Size and Surface Functionality on the Cellular Uptake of Sub-10 nm Gold Nanoparticles. *ACS Nano* **2015,** *9* (10), 9986-93.
14. Matus, M. F.; Häkkinen, H., Rational Design of Targeted Gold Nanoclusters with High Affinity to Integrin αvβ3 for Combination Cancer Therapy. *Bioconjugate Chemistry* **2024,** *35* (10), 1481-1490.
15. Zhao, J. Y.; Cui, R.; Zhang, Z. L.; Zhang, M.; Xie, Z. X.; Pang, D. W., Cytotoxicity of nucleus-targeting fluorescent gold nanoclusters. *Nanoscale* **2014,** *6* (21), 13126-34.

16. Zhuang, Q.; Jia, H.; Du, L.; Li, Y.; Chen, Z.; Huang, S.; Liu, Y., Targeted surface-functionalized gold nanoclusters for mitochondrial imaging. *Biosens Bioelectron* **2014,** *55*, 76-82.
17. Desplancq, D.; Groysbeck, N.; Chiper, M.; Weiss, E.; Frisch, B.; Strub, J.-M.; Cianferani, S.; Zafeiratos, S.; Moeglin, E.; Holy, X.; Favier, A. L.; De Carlo, S.; Schultz, P.; Spehner, D.; Zuber, G., Cytosolic Diffusion and Peptide-Assisted Nuclear Shuttling of Peptide-Substituted Circa 102 Gold Atom Nanoclusters in Living Cells. *ACS Applied Nano Materials* **2018,** *1* (8), 4236-4246.
18. Melkikh, A.; Sutormina, M., Model of active transport of ions in cardiac cell. *Journal of theoretical biology* **2008,** *252*, 247-54.
19. Reinert, A.; Morawski, M.; Seeger, J.; Arendt, T.; Reinert, T., Iron concentrations in neurons and glial cells with estimates on ferritin concentrations. *BMC Neurosci* **2019,** *20* (1), 25.
20. Babak, M. V.; Ahn, D., Modulation of Intracellular Copper Levels as the Mechanism of Action of Anticancer Copper Complexes: Clinical Relevance. *Biomedicines* **2021,** *9* (8).
21. Falcone, E.; Okafor, M.; Vitale, N.; Raibaut, L.; Sour, A.; Faller, P., Extracellular Cu2+ pools and their detection: From current knowledge to next-generation probes. *Coordination Chemistry Reviews* **2021,** *433*.
